# Loss-of-Function Mutations in *CsMLO1* Confer Durable Powdery Mildew Resistance in Cucumber (*Cucumis sativus* L.)

**DOI:** 10.3389/fpls.2015.01155

**Published:** 2015-12-22

**Authors:** Jingtao Nie, Yunli Wang, Huanle He, Chunli Guo, Wenying Zhu, Jian Pan, Dandan Li, Hongli Lian, Junsong Pan, Run Cai

**Affiliations:** ^1^School of Agriculture and Biology, Shanghai Jiao Tong UniversityShanghai, China; ^2^College of Agronomy, Heilongjiang Bayi Agricultural UniversityDaqing, China

**Keywords:** cucumber, powdery mildew, durable resistance, *MLO*, loss of function, transcriptome

## Abstract

Powdery mildew (PM) is a serious fungal disease of cucumber worldwide. The identification of resistance genes is very important for resistance breeding to ensure cucumber production. Here, natural loss-of-function mutations at an *MLO* homologous locus, *CsMLO1*, were found to confer durable PM resistance in cucumber. *CsMLO1* encoded a cell membrane protein, was mainly expressed in leaves and cotyledons, and was up-regulated by PM at the early stage of host–pathogen interaction. Ectopic expression of *CsMLO1* rescued the phenotype of the PM resistant *Atmlo2 Atmlo12* double mutant to PM susceptible in *Arabidopsis*. Domesticated and wild resistant cucumbers originating from various geographical regions of the world were found to harbor three independent natural mutations that resulted in *CsMLO1* loss of function. In addition, between the near-isogenic lines (NILs) of PM resistant and susceptible, S1003 and NIL(*Pm5.1*), quantitative RT-PCR revealed that there is no difference at expression levels of several genes in the pathways of ethylene, jasmonic acid or salicylic acid. Moreover, the two NILs were used for transcriptome profiling to explore the mechanism underlying the resistance. Several genes correlated with plant cell wall thickening are possibly involved in the PM resistance. This study revealed that loss of function of *CsMLO1* conferred durable PM resistance, and that this loss of function is necessary but alone may not be sufficient for PM resistance in cucumber. These findings will facilitate the molecular breeding of PM resistant varieties to control this destructive disease in cucumber.

## Introduction

Cucumber (*Cucumis sativus* L.) is an economically important crop cultivated worldwide (Huang et al., 2009). Powdery mildew (PM) is a common disease of cucumber under field and greenhouse conditions globally, and is a major threat to cucumber farming with regard to both product yield and quality. Two pathogens, *Podosphaera xanthii* (previously known as *Sphaerotheca fuliginea*) and *Golovinomyces cichoracearum* (formerly *Erysiphe cichoracearum*), have been reported as the most common fungi causing cucumber PM, with the former being predominant in most countries (Bardin et al., 1999; Hosoya et al., 1999; Del Pino et al., 2002; Pérez-García et al., 2009). So far, the application of protective fungicides has been the major way of controlling this disease. However, PM has become resistant to chemical fungicides used over many years. In addition, there has been greater focus on environment protection and public health recently. Thus, breeding cultivars resistant to PM is the most effective and environment-friendly strategy for controlling this disease.

At least for obligate pathogens such as PM, the majority of resistance genes deliberately deployed by breeders are race-specific resistance (R) genes (Collins et al., 2007). However, because R-genes confer race-specific resistance, which can easily be overcome by new pathogen races in a short period, *R* gene resistance to PM is of limited agronomic value. In contrast, the loss-of-function *mlo* (*mildew resistance locus o*) conferred almost complete, durable, and broad-spectrum resistance to PM in the monocot barley and the dicots *Arabidopsis*, tomato and pea (Büschges et al., 1997; Consonni et al., 2006; Bai et al., 2008; Humphry et al., 2011). Because of its effectiveness and longevity, *mlo* resistance has been successfully used in European agriculture for several decades with hardly any breakdown in effectiveness (Jørgensen, 1992; Lyngkjær et al., 1995).

*MLO* genes encode a novel type of plant-specific integral membrane protein with an as yet unknown basic biochemical mode of action (Devoto et al., 1999; Panstruga, 2005a). The genes comprise small to medium-size families that vary between plant species (Devoto et al., 2003; Liu and Zhu, 2008; Konishi et al., 2010). In contrast to barley, where functional specialization for PM resistance suppression appears to be complete and confined to one *MLO* member, three *MLOs* (*AtMLO2*, *AtMLO6*, and *AtMLO12*) contribute to resistance suppression in *Arabidopsis*, with *AtMLO2* playing a predominant role (Consonni et al., 2006; Collins et al., 2007). The *mlo* allele acts early and confers pre-invasion resistance by terminating fungal pathogenesis before plant cell entry during the process of cell wall penetration (Jørgensen, 1992; Lyngkjær et al., 2000). The barley MLO protein modulates the defense response at the cell periphery via a vesicle-associated and SNARE protein-dependent mechanism, and the PM pathogen possibly exploits these proteins for successful host cell entry (Panstruga, 2005b). An MLO-homolog was identified in a screen for rice proteins that interact with calmodulin, prompting the examination of the role of calmodulin in *mlo* resistance in barley (Kim et al., 2002a,b). Kim et al. (2002b) showed that the MLO protein functions independently of heterotrimeric G proteins and mediates a Ca^2+^-dependent interaction with calmodulin *in vitro*. Loss of calmodulin binding halves the ability of MLO to negatively regulate defense against PM *in vivo*. In *Arabidopsis*, *mlo* resistance does not involve the signaling molecules ethylene, jasmonic acid (JA) or salicylic acid (SA), but requires a syntaxin, glycosyl hydrolase and ATP-binding cassette (ABC) transporter to limit invasion by PMs (Collins et al., 2003; Lipka et al., 2005; Consonni et al., 2006; Stein et al., 2006).

Although *mlo*-mediated PM resistance has been studied in the monocot barley and the dicots *Arabidopsis thaliana*, tomato and pea, the genes and mechanisms underlying the PM resistance in cucumber and other cucurbits remain unknown. Genetic analysis demonstrated that PM resistance in cucumber was quantitatively inherited and controlled by major recessive genes, and QTL analysis was performed to find the genetic factors affecting resistance (Sakata et al., 2006; Liu et al., 2008a,b; Zhang et al., 2011; Fukino et al., 2013; He et al., 2013). Recently, two recessive major genes/QTLs for PM resistance were identified in the same region on chromosome 5 (He et al., 2013; Nie et al., 2015) and one *MLO*-like gene was identified as the candidate for the major locus *pm5.1* through map-based cloning (Nie et al., 2015). In this study, this *MLO*-like gene (designated *CsMLO1*) was confirmed to mediate PM resistance in cucumber through several experiments. Moreover, the mechanism of *pm5.1*-mediated resistance in cucumber was preliminarily explored, and found to may not involve the signaling molecules ethylene, JA or SA. Additionally, several possible key genes, involved in plant cell wall defense and encoding ABC transporters, were identified as involved in *pm5.1*-mediated resistance through transcriptome profiling, which provides a solid foundation for further studies on the mechanism of PM resistance in cucumber. The present study on PM resistance in cucumber should facilitate molecular breeding for disease resistant varieties.

## Materials and Methods

### Plant and Fungal Materials

The 28 cucumber inbred lines used for sequencing of *CsMLO1* alleles in this study are listed in Supplementary Table S1. Among them, lines S1003, S1001, and S05 were previously used to map the major locus *pm5.1* for PM resistance (Nie et al., 2015). Two near-isogenic lines (NILs), S1003 and NIL(*Pm5.1*), were used for qRT-PCR and transcriptome profiling analysis. NIL(*Pm5.1*) is an introgression line in which a small S05 chromosomal segment containing the *Pm5.1* (a *pm5.1* allele) locus was introduced into the S1003 genetic background. S1003 has been highly resistant to PM for several decades while NIL(*Pm5.1*) is highly susceptible. The *Arabidopsis* double mutant *mlo2-5 mlo12-1* (CS9713^1^) described previously (Consonni et al., 2006) was provided by the *Arabidopsis* Biological Resource Center. The PM pathogen (*P. xanthii*) used was isolated from the greenhouse in Shanghai Jiao Tong University, China, and maintained by infection of susceptible cucumber line S05 as described previously (Nie et al., 2015).

### Sequencing of *CsMLO1* Alleles

According to the cDNA sequence predicted using the FGENESH program^2^, specific primers (Supplementary Table S2) were designed to amplify the cDNA sequence of the *pm5.1* candidate gene *CsMLO1*. The PCR fragments amplified from the cDNAs were cloned into TA vectors and were sequenced. The promoter region of *CsMLO1* in lines S1003, S1001 and S05 was sequenced (Supplementary Table S2). The PlantCARE database^3^ was used to analyze the *cis*-acting regulatory elements in the *CsMLO1* promoter sequences (Lescot et al., 2002). Specific primers (Supplementary Table S2) were used to amplify the DNA sequences of the *CsMLO1* alleles from different cucumber inbred lines. The coding sequences (CDSs) and amino acid sequences of the *CsMLO1* alleles were predicted by FGENESH. Alignment of the DNA and amino acid sequences of *CsMLO1* alleles from different cucumber lines was performed using the DNAMAN software^4^.

### Phylogenetic Analysis

The deduced amino acid sequence of the CsMLO1 protein was compared with those of MLOs from barley, maize, *Arabidopsis*, tomato and pea. The protein sequences were aligned using the default settings in the DNAMAN software. Phylogenetic analyses at the amino acid level were performed using the DNAMAN software with the parameters “observed divergency” and “toss gaps”, and the support for the branching arrangements was evaluated by bootstrap analysis using 1000 replicates. The GenBank accession numbers of *Arabidopsis* AtMLO1–AtMLO15, barley HvMLO (Z83834), and maize ZmMLO1 (AY029312) came from Devoto et al. (2003). The other MLOs used were tomato SlMLO1 (AY967408) and pea PsMLO1 (FJ463618). The predicted MLO proteins in the cucurbit crops cucumber, watermelon and melon were screened from the cucurbit genomics database^5^ (Huang et al., 2009; Guo et al., 2013) and melonomics (Garcia-Mas et al., 2012).

### Subcellular Localization of *CsMLO1*

A preliminary analysis of the transmembrane structure of the CsMLO1 protein was conducted using the online transmembrane structure prediction tools TOPCONS^6^ and TMHMM^7^. The CDS of *CsMLO1* was cloned into the pHB-GFP vector between the *SpeI* and *Sal*I sites. The *GFP-CsMLO1* fusion was driven by the 35S promoter. The control vector *pHB-GFP* and the *Cauliflower mosaic virus* (CaMV) *35S::GFP-CsMLO1* fusion construct were infiltrated into tobacco leaf epidermal cells using the *Agrobacterium*-mediated method (Sparkes et al., 2006). Samples were observed with a Leica TCS SP5-II confocal fluorescence microscope. The primers for vector construction are listed in Supplementary Table S2.

### Complementation Test of *CsMLO1* in *Arabidopsis*

The *CsMLO1* over-expression construct was the same as that used for subcellular localization analysis. The recombinant plasmids were introduced into *Agrobacterium* by electroporation and then transformed into *mlo2-5 mlo12-1* double mutant (CS9713) plants through the floral-dip method (Clough and Bent, 1998). The transgenic plants were screened on 1/2 MS medium with 50 mg/L hygromycin. The primers for transgenic plant detection are listed in Supplementary Table S2. GFP in the positive transgenic plants was observed with a Leica confocal fluorescence microscope. The controls Col-0 (wild type) and CS9713 and the transgenic plants were inoculated with *Arabidopsis* PM, and their infection phenotypes were observed at 12 days post inoculation.

### DNA and RNA Extraction and Gene Expression Analysis

Cucumber genomic DNA was extracted using the CTAB method (Murray and Thompson, 1980). For gene expression analysis, total RNA was prepared from different samples and genomic DNA was eliminated with an RNAprep pure Plant Kit (TIANGEN, China), according to the manufacturer’s instructions. First-strand cDNA was synthesized using the PrimeScript first Strand cDNA Synthesis Kit (Takara, Japan). Gene expression was analyzed by semi-quantitative RT-PCR and quantitative RT-PCR (qRT-PCR). qRT-PCR was carried out using the SYBR *Premix Ex Taq* II Kit (Takara, Japan) and the PCR amplification was quantified according to the manufacturer’s protocol. Amplification was performed on a CFX96^TM^ real-time system (Bio-Rad, USA). The *actin* gene of cucumber (*CsActin*) was used to quantify the relative transcript levels. The expression of each gene was calculated by the comparative Ct method (Simon, 2003), based on the relative expression of the target gene versus the reference gene *CsActin*. To examine the tissue-specific expression patterns of *CsMLO1*, different cucumber organs of lines S1003, S1001 and S05 were sampled. For expression modeling of the *CsMLO1* gene interaction with the PM pathogen and to identify signaling and resistance pathway genes potentially involved in PM resistance in cucumber, the NILs S1003 and NIL(*Pm5.1*) were grown under axenic conditions in a growth chamber at 25°C/22°C (day/night) with a 16/8-h (light/dark) photoperiod. At the third-leaf stage, the plants were inoculated with the pathogen by spraying a spore suspension (1 × 10^5^ spores/ml) evenly onto the leaves and sampled before inoculation (0 h) and after 3, 6, 12, 24, 48, and 72 h. Each experiment was sampled with three biological replicates. All primers used in this analysis are listed in Supplementary Table S2.

### Transcriptome Analysis

Transcriptome profiling experiments were carried out using the digital gene expression (DGE) approach. The plants for transcriptome analysis were grown and inoculated under severe and consistent conditions. S1003 and NIL(*Pm5.1*) were cultivated and inoculation was carried out as described above. The transcriptomes of leaves (before inoculation and 12 h post inoculation (hpi)) from the two NILs (each sample was mixed by three individuals) were sequenced on the Illumina HiSeq 2500 platform. Total RNA was extracted from the samples using TRIzol reagent (Invitrogen, USA), and treated with RNase-free DNase (Takara, Japan). The total RNA was checked for both quality and quantity, and only paired RNA of high quality was used for subsequent analyses. RNA-seq libraries were prepared and sequenced at Genergy (Shanghai, China). For each gene, the expression level was calculated from the baseMean value, which was the sequencing depth for each transcript normalized to the library size, and was measured in “Fragments Per Kilobase of transcript per Million fragments mapped” (FPKM). RNA-seq reads were counted against cucumber gene annotations using HTSeq^8^. Normalization and differential gene expression analysis were performed with DESeq^9^. Cucumber gene IDs refer to the published cucumber genome database^10^ (Huang et al., 2009). Genes with at least a two-fold change in expression and a Benjamini and Hochberg-adjusted *P*-value less than 0.05 were considered differentially expressed. The genes were annotated by reference to the cucumber database^11^.

The genes expressed in the four cucumber leaf libraries, i.e., S1003 before inoculation (R0), S1003 at 12 hpi (R12), NIL(*Pm5.1*) before inoculation (S0), NIL(*Pm5.1*) at 12 hpi (S12) were separately investigated by DGE analysis. Three groups of differentially expressed genes (DEGs), i.e., R12 vs. R0, S12 vs. S0 and R12 vs. S12, were analyzed. Venn analysis was performed using the Venny 2.0 program^12^. Gene ontology (GO) enrichment analysis was used to identify all the GO terms that were significantly enriched in the DEGs compared with the genomic background. The DEGs corresponding to biological functions were filtered.

## Results

### *CsMLO1* Cloning with a PCR-Based Approach

**FIGURE 1:**
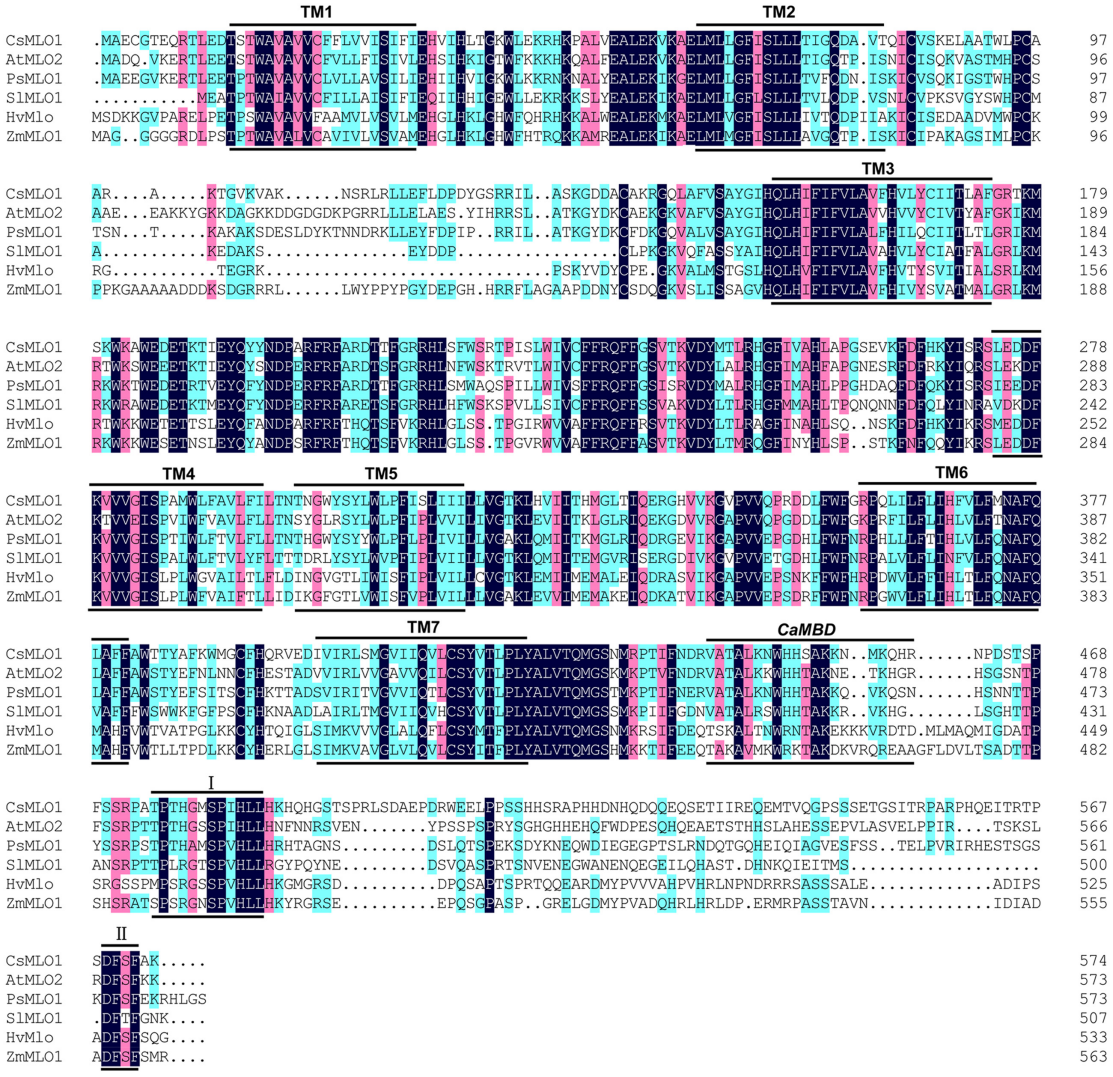
**Multiple amino acid sequence alignment of CsMLO1 and other MLO proteins**. The amino acid sequences of HvMLO (barley), AtMLO2 (*Arabidopsis*), ZmMLO1 (maize), SlMLO1 (tomato), PsMLO1 (pea), and CsMLO1 (cucumber) were aligned with DNAMAN using the default parameters. The positions of the transmembrane regions (TM1–TM7) inferred from the experimentally determined topology of barley HvMLO (Devoto et al., 1999), the approximate position of the CaMBD (Kim et al., 2002a,b) and the other two conserved domains (I and II; Panstruga, 2005b) are indicated by lines above and below the aligned sequences.

In a previous study, one major locus on chromosome 5, *pm5.1*, conferring PM resistance was identified and one *MLO*-like gene (*CsMLO1*) was characterized as the candidate for *pm5.1* through map-based cloning. A 1449-bp DNA fragment was inserted in the 11th exon of the *CsMLO1* gene in S1003. The recessive nature of the PM resistance mediated by *pm5.1* might be caused by loss of function of *CsMLO1* in S1003 (Nie et al., 2015). The *CsMLO1* gene was characterized through a PCR-based approach and TA cloning. Its open reading frame (ORF) was 1725 bp and the deduced peptide consisted of 574 amino acid residues. The CsMLO1 protein shared highly similarity with proteins encoded by *MLO* genes for PM susceptibility in barley, *Arabidopsis*, tomato and pea (Büschges et al., 1997; Consonni et al., 2006; Bai et al., 2008; Humphry et al., 2011) (**Figure 1**). CsMLO1 shared 59.28, 53.22, 59.80, 42.47, and 44.52% homology with *Arabidopsis* AtMLO2, tomato SlMLO1, pea PsMLO1, barley HvMLO, and maize ZmMLO1, respectively. All of the proteins were highly conserved at the predicted seven transmembrane domains (TM1–TM7; Devoto et al., 1999), the CaMBD position (Kim et al., 2002a,b) and the other two conserved domains (I and II) in the highly polymorphic C-terminus (Panstruga, 2005b), (**Figure 1**).

Sequence comparison of 2 kb of the putative *CsMLO1* promoter regions revealed near identical sequences among the parental lines S1003, S1001 and S05 (Supplementary Figure S1). The *cis*-acting regulatory elements in the *CsMLO1* promoter sequences were analyzed. Fungal elicitor responsive and defense and stress responsive elements were found in putative promoter regions, indicating that *CsMLO1* might participate in defense responses to PM fungal infestation (Supplementary Table S3).

**FIGURE 2:**
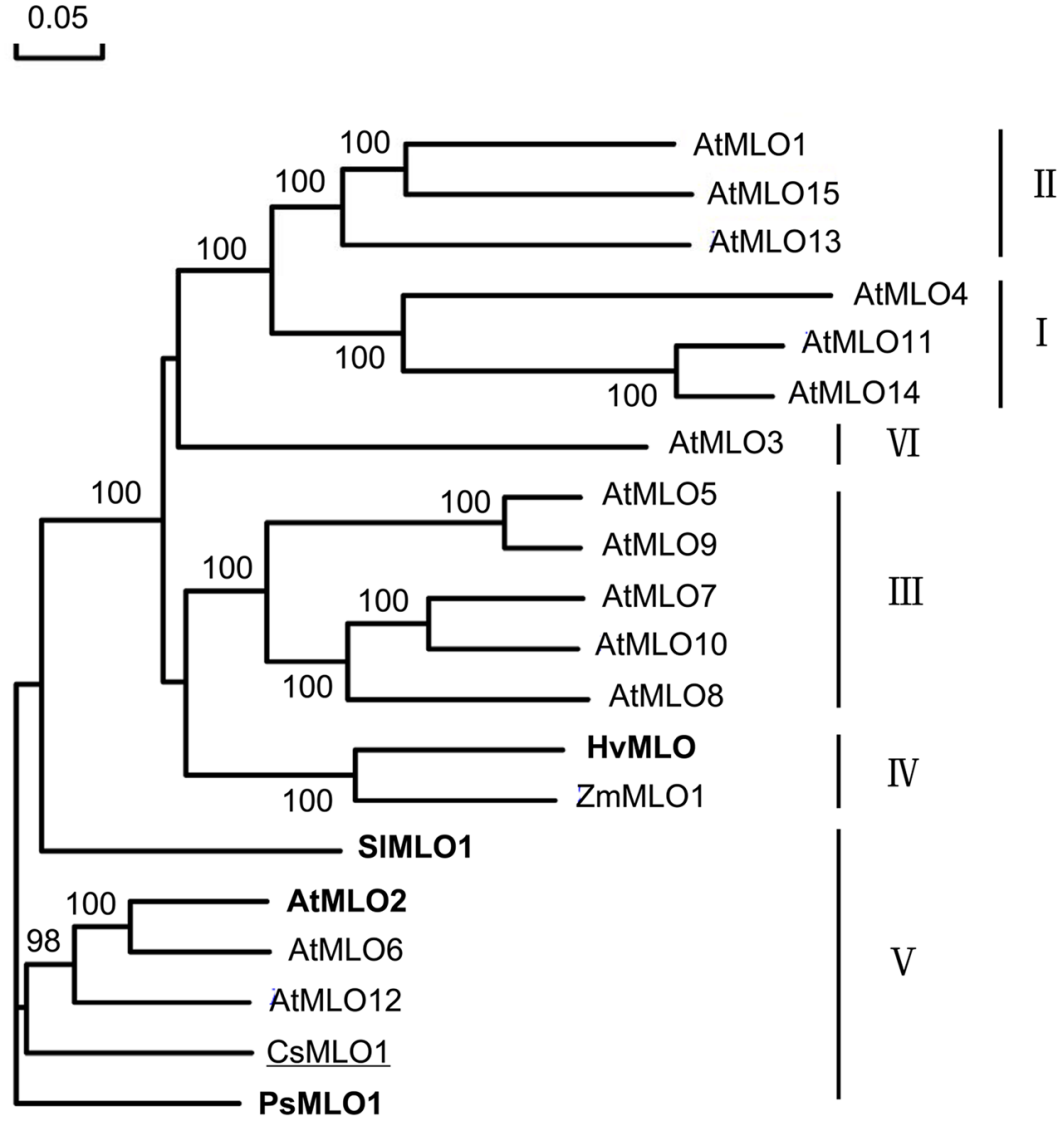
**Phylogenetic relationships of CsMLO1 and MLO proteins from other species, including HvMLO (barley), AtMLO1–AtMLO15 (*Arabidopsis*), ZmMLO1 (maize), SlMLO1 (tomato) and PsMLO1 (pea)**. The six clades were designated according to Devoto et al. (2003). Proteins that have been proven to play an important role in powdery mildew (PM) resistance are highlighted in bold; cucumber CsMLO1 is underlined. Numbers at the nodes indicate bootstrap support based on 1000 replicates.

Phylogenetic analysis of CsMLO1 and other MLO protein sequences from dicots and monocots revealed that the CsMLO1 protein was in the dicot clade V with AtMLO2, AtMLO6, AtMLO12, SlMLO1, and PsMLO1, and that HvMLO was in the monocot clade IV (**Figure 2**); each of these proteins has been shown previously to be required for PM susceptibility (Büschges et al., 1997; Consonni et al., 2006; Bai et al., 2008; Humphry et al., 2011). This finding and the presence of a D-F-S-F tetrapeptide motif at the C-terminus of the proteins (**Figure 1**), which is considered to be diagnostic of an orthologous phylogenetic relationship (Panstruga, 2005b), suggested CsMLO1 was a co-ortholog of the monocot barley HvMLO and the dicot *Arabidopsis* AtMLO2, AtMLO6, and AtMLO12 proteins. These results strongly suggested that *CsMLO1* might be *Pm5.1*, which negatively regulates PM resistance in cucumber.

### Subcellular Localization and Expression Pattern of *CsMLO1*

**FIGURE 3:**
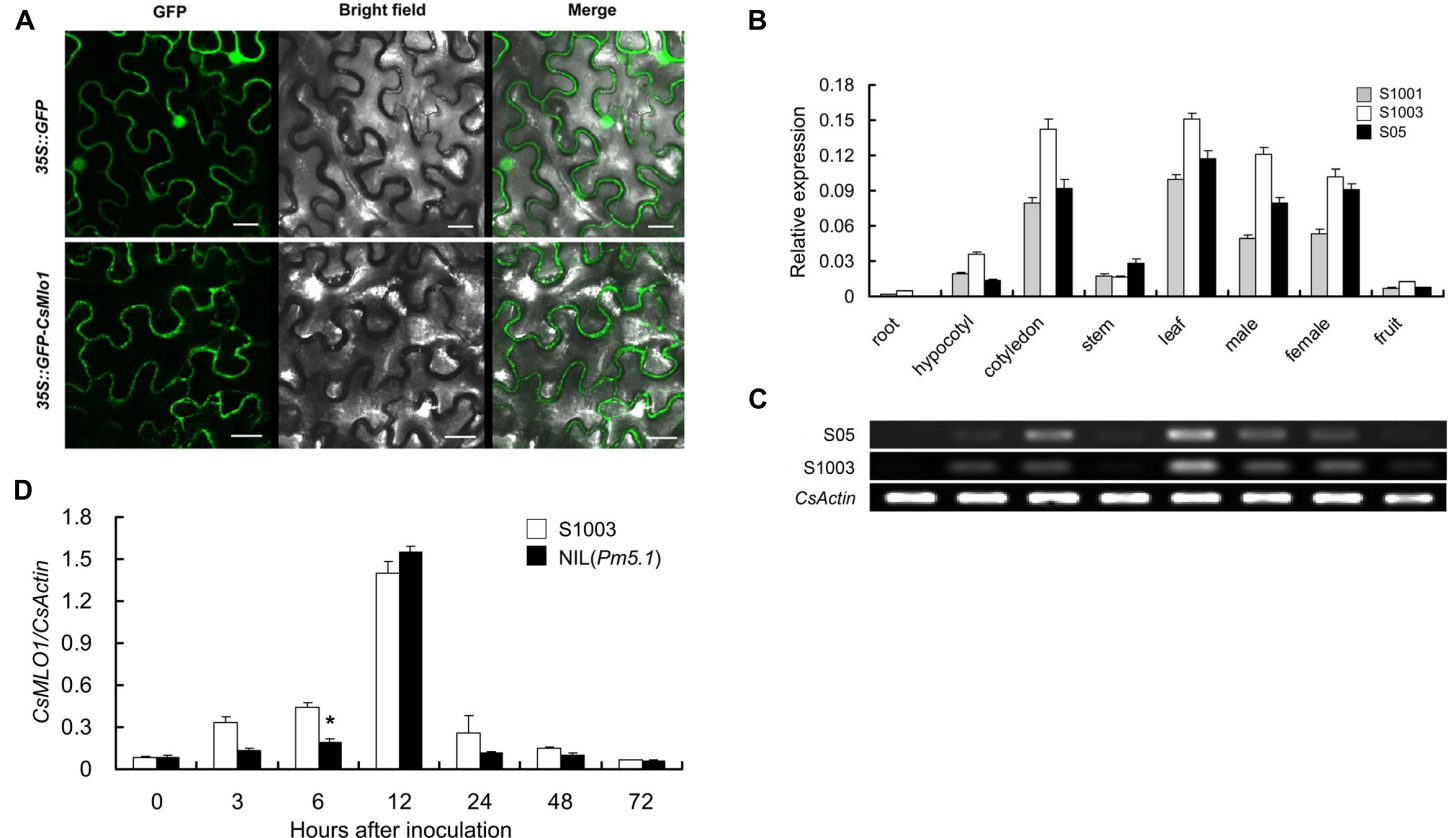
**Subcellular localization and expression pattern of *CsMLO1***. **(A)** *35S::GFP* and *35S::GFP-CsMLO1* constructs were transiently expressed in tobacco leaf epidermal cells. The fusion protein GFP-CsMLO1 was localized in the cell membrane. Scale bars = 20 μm. **(B,C)** Spatial expression patterns of *CsMLO1* alleles in roots, hypocotyls, cotyledons, stems, leaves, male flowers, female flowers, and fruits of S1001, S1003, and S05 according to qRT-PCR **(B)** and semi-quantitative RT-PCR analyses **(C)**. *S05* and *S1003* indicate the *CsMLO1* allele expression in cucumber lines S05 and S1003, respectively. **(D)** Time-dependent expression pattern of *CsMLO1* in resistant S1003 and susceptible NIL(*Pm5.1*) plants after PM inoculation. The cucumber *CsActin* gene was used to quantify the relative transcript levels of *CsMLO1*. Values are mean ± SE (*n* = 3) (^∗^indicates significant difference between S1003 and NIL(*Pm5.1*) at the *P* = 0.05 level).

The transmembrane structure of the protein encoded by *CsMLO1* was first analyzed using online tools. CsMLO1 possessed typical characteristics of the MLO family, with seven transmembrane structures (Supplementary Figure S2). The subcellular localization of the CsMLO1 protein was then analyzed. Confocal imaging showed that the fusion protein GFP-CsMLO1 was localized exclusively in the cell membrane in a transient expression assay (**Figure 3A**). In the control, the GFP protein was found in the cell membrane, cytoplasm, and nucleus.

To investigate the spatial expression patterns of *CsMLO1*, semi-quantitative RT-PCR and quantitative RT-PCR analyses were conducted with total RNA extracted from different cucumber organs of S1003, S1001, and S05. *CsMLO1* exhibited relatively high expression in leaves, cotyledons, and male and female flowers, but faint expression in hypocotyls and stems, and was barely expressed in roots and fruits (**Figures 3B,C**).

In addition, the expression of *CsMLO1* was enhanced by PM inoculation and was significantly different only at 6 hpi between S1003 and NIL(*Pm5.1*) (**Figure 3D**). These results indicated that the sequence variations in the coding region, not the allelic transcription levels, accounted for the gene being functional in PM resistance/susceptibility.

### Complementation Test of *CsMLO1* in *Arabidopsis*

**FIGURE 4:**
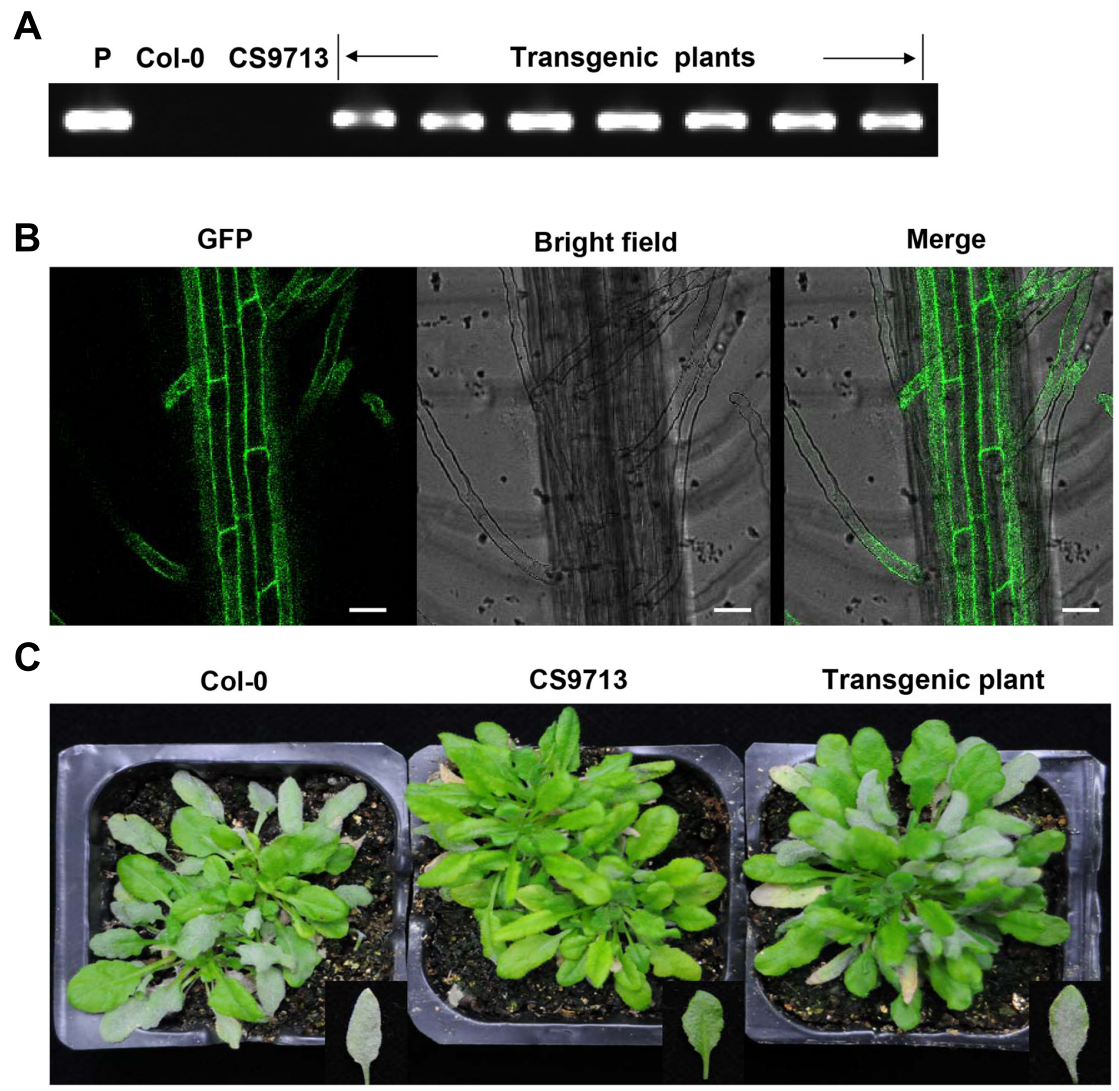
**Complementation test of CsMLO1 in *Arabidopsis***. **(A)** PCR results for the recombinant plasmid, the Col-0 wild type, the *Atmlo2 Atmlo12* double mutant CS9713 and the transgenic plants. **(B)** GFP fluorescence in the roots of the transgenic plants in the *Arabidopsis* CS9713 genetic background. Scale bars = 30 μm. **(C)** Infection phenotype of the Col-0 wild type, the *Atmlo2 Atmlo12* double mutant CS9713 and the transgenic plants at 12 days post-inoculation.

To further confirm that *CsMLO1* was *Pm5.1*, the defect in *mlo Arabidopsis* plants was complemented by over-expressing C*sMLO1* in the *mlo2-5 mlo12-1* mutant (CS9713) in the *Columbia* (*Col*) background. A total of seven independent transgenic lines were obtained. PCR and GFP observation showed that the T-DNA construct was integrated into the genome of the transgenic plants and the fusion protein GFP-CsMLO1 was expressed and localized in the cell membrane (**Figures 4A,B**). The transgenic plants exhibited enhanced PM susceptibility in contrast with the mutant (**Figure 4C**). These results indicated that *CsMLO1* was able to complement the susceptibility phenotype in *Arabidopsis*. Therefore, the results further supported the conclusion that *CsMLO1* corresponded to *Pm5.1*. The results also showed that the functions of *CsMLO1*, *AtMLO2*, and *AtMLO12* were conserved in the course of evolution following the divergence of these two species, in accordance with the conclusion inferred from phylogenetic analysis (**Figure 2**).

### *CsMLO1* is Defective in Three Independent Natural Mutation Types

**FIGURE 5:**
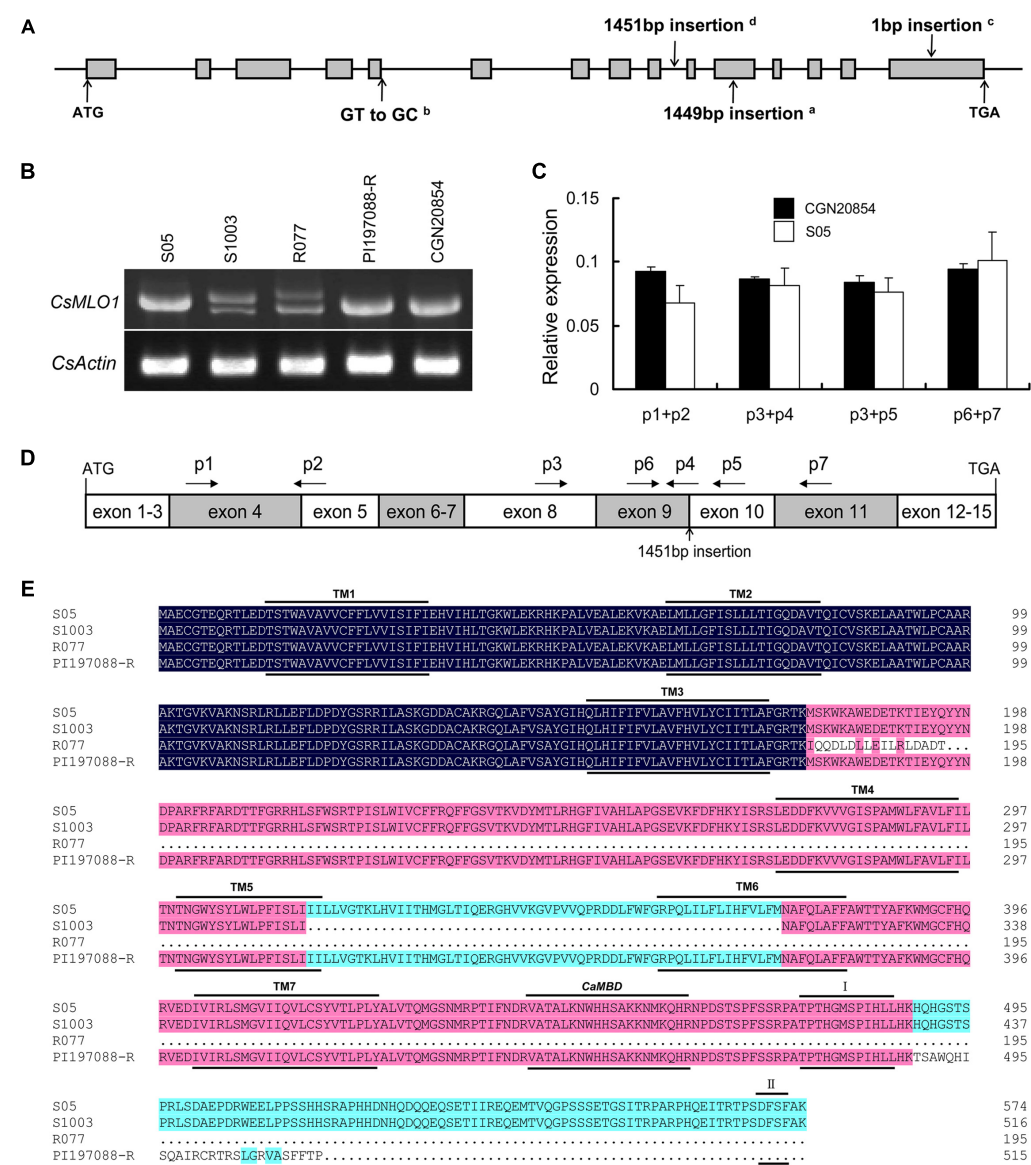
**Variant alleles of CsMLO1 and their effects on the transcripts and proteins**. **(A)** Gene structure and haplotype analysis of *CsMLO1*. Gene structure is shown using the *CsMLO1* gene sequence of S05 as a reference. Gray boxes and lines between them denote exons and introns, respectively. The mutation types in the different resistant cucumber lines and susceptible line CGN20854 are indicated by arrows. ^a^The 1449-bp insertion mutation in the S1003-like haplotype (haplotype A); ^b^the splicing site mutation from GT to GC in the R077-like haplotype (haplotype B); ^c^the 1-bp insertion mutation in PI197088-R (haplotype C); ^d^the 1451-bp insertion mutation in CGN20854. ATG, start codon; TGA, stop codon. **(B)** Identification of transcript variations by expression analysis via RT-PCR, focusing on the entire coding transcript. **(C,D)** Confirmation of the expression levels of the *CsMLO1* allele in line CGN20854 by qRT-PCR using different primer combinations. **(C)** qRT-PCR analysis of *CsMLO1* in line CGN20854; S05 was used as a control. Leaves were sampled at the seedling stage for analysis. The cucumber *CsActin* gene was used to quantify the relative expression level of *CsMLO1*. Values are mean ± SE (*n* = 3). **(D)** Scheme describing the relative positions and orientations of primers (p1–p7) with respect to the CDS of *CsMLO1* in CGN20854. Boxes denote exons. The 1451-bp insertion mutation at the nineth intron of the *CsMLO1* allele in CGN20854 is indicated by an arrow. **(E)** Amino-acid sequence alignment of different haplotypes. The seven transmembrane regions (TM1–TM7), the approximate CaMBD position and the other two conserved domains (I and II) are indicated by lines above and below the aligned sequences.

To further confirm that *CsMLO1* was an allele of *pm5.1*, the sequences of the *CsMLO1* alleles in 28 cucumber inbred lines (resistant or susceptible to PM) originating from various geographical regions of the world were analyzed (Supplementary Table S1). Among them, WI2757 and True Lemon had been used for QTL analysis of PM resistance previously. A major QTL (*pm5.2*) was identified in WI2757, located in the same chromosome region as *pm5.1* in S1003 (He et al., 2013; Nie et al., 2015). PI197088-R and PI197088-S, the progeny of the wild type line PI197088, which originated in India, are resistant and susceptible to PM, respectively (Supplementary Table S1). Through comparison of the coding regions among *CsMLO1* alleles, most of the susceptible cucumber lines were found to have the same or almost identical sequences as S1001 and S05, and all of their predicted protein sequences were almost identical (Supplementary Figures S3A,B), while three independent natural mutation types occurred in the coding regions of the *CsMLO1* alleles of the resistant lines leading to splice site mutations resulting in mis-splicing, frame shifts, or premature stop codons (**Table 1**). Most of the resistant cucumber lines, including S1003 and WI2757, had an S1003-like haplotype (haplotype A) with a 1449-bp DNA fragment insertion at nucleotide position 2799 in the 11th exon of *CsMLO1*; four resistant cucumber lines shared an R077-like haplotype (haplotype B) harboring a GT-to-GC splice-site variation at nucleotide position 1301 at the beginning of the fifth intron; and one resistant line had haplotype C with a 1-bp insertion at nucleotide position 3703, in the 15th exon of the gene (**Table 1**; **Figure 5A**). There were three exceptions in the susceptible lines CGN20854, 9930 and S94, which had a 1451-bp DNA fragment insertion at nucleotide position 2581 in the nineth intron, an R077-like haplotype and an S1003-like haplotype but with a 1467-bp DNA fragment insertion at the same mutation position, respectively (**Figure 5A**).

Interestingly, the sequences of the insertion fragments of haplotype A and CGN20854 were nearly identical (95.6% identity), with one short fragment duplication (Target Site Duplication, TSD) 5′-ATTAT-3′ and 5′-CCTTT-3′ flanking both sides of the insertions of haplotype A and CGN20854, respectively (Supplementary Figure S4A). There were 184 bp and 196 bp identical long terminal repeats (LTRs) at both ends of the insertion sequences (after and before the TSD) of haplotype A and CGN20854, respectively (Supplementary Figure S4B). Moreover, BLASTN analysis of the insertion sequences in TAIR^13^ was performed and the best hit was a transposable element gene, *AT3G30815.1*, which is a member of *Copia*-like retrotransposon family. Therefore, the results showed that the function of *CsMLO1* was disturbed via insertion mutations in haplotype A, by integration of a transposable element (TE) similar to the *Copia* LTR-retrotransposon.

To determine whether the cDNAs of the *CsMLO1* alleles were changed by the three mutation types in resistant cucumber lines and by the mutation in susceptible line CGN20854, expression analysis was performed using RT-PCR, focusing on the entire coding transcript. Compared with the susceptible line S05, the S1003 and R077 lines showed low levels of truncated transcripts, whereas lines PI197088-R and CGN20854 showed similar bands to S05 (**Figure 5B**). Sequence analysis of the transcripts of S1003, R077, PI197088-R, and CGN20854 further showed that the entire 11th exon (174 bp) and fifth exon (61 bp) were lost in S1003 and R077, respectively, the transcript of PI197088-R had a 1-bp insertion compared with that of S05 at the 15th exon, and the transcript of CGN20854 shared the same 1725-bp CDS as that of the susceptible line S52, which had 1-bp difference from that of S1001 (Supplementary Figure S5A). The cDNA of the *CsMLO1* allele in 9930 was also sequenced and was the same as that of R077 (Supplementary Figure S5A). Additionally, three other cDNAs were obtained by transcriptome analysis for S1003 and one of them stemmed from the insertion of the 1449-bp DNA fragment into the *CsMLO1* genomic sequence (Supplementary Figure S5B). Although CGN20854 shared a similar insertion sequence to S1003 in the coding region of *CsMLO1*, the insertion event in the nineth intron did not influence the normal expression of the transcript, which was confirmed by qRT-PCR using different primer combinations (**Figures 5C,D**). The truncated transcript in S1003 (haplotype A) encoded a protein lacking amino acids 316–373, which contained part of the fifth and sixth transmembrane regions and the intracellular loop ring between them, whereas the truncated transcript in R077 (haplotype B) had a frame shift that resulted in the introduction of a premature stop codon, and therefore encoded a protein lacking the normal amino acids from position 179 (**Figure 5E**). The 1-bp insertion in the transcript of PI197088-R (haplotype C) also resulted in the introduction of a premature stop codon, and the encoded protein lacked the normal amino acids from position 489 (**Figure 5E**).

An allelism test was performed using three resistant haplotype lines (Supplementary Table S4). The results showed that the loci conferring PM resistance in R077 and PI197088-R were allelic to *pm5.1* in S1003. In addition, PI197088-R is a spontaneous mutant from wild cucumber PI197088, and the recessive nature of its resistance is in accordance with the loss of function of the *CsMLO1* allele (**Figure 5**; Supplementary Table S4). Furthermore, an identical mutation event in *CsMLO1* occurred in S1003 and WI2757, which together with *pm5.2* and *pm5.1* being located in the same chromosome region, indicated that *pm5.2* might be an allele of *pm5.1* (He et al., 2013; Nie et al., 2015). The identification of three independent natural mutation events of *CsMLO1* alleles in PM-resistant lines (with *pm5.1*-mediated or unknown resistance) of distinct geographical origin strongly indicated that loss of function of *CsMLO1* was responsible for conferring the *pm5.1*-mediated resistance in cucumber.

**Table 1:** Molecular characterization of the three independent natural mutation types of the *CsMLO1* alleles in resistant cucumber lines.

Mutation event	Position of mutation (bp)^∗^	Effect of mutation	Haplotype	Number of lines
Insertion of 1449 bp	2799, at the 11th exon	Truncated transcript missing exon 11; other transcripts	A	10
GT→GC	1301, at the beginning of the fifth intron	Mis-splicing, truncated transcript without exon 5	B	4
1 bp insertion	3703, at the 15th exon	Frame shift after amino acid 488 and premature stop codon at amino acid 515	C	1

*^∗^Based on the position of the genomic sequence of the *CsMLO1* allele of S05*.

### *MLO* Family Members in Cucurbits

Predicted MLO family proteins in the cucurbit crops cucumber, melon and watermelon were identified by searching the cucurbit genomics database. Twelve, 15, and 11 predicted MLO proteins were found in cucumber, melon and watermelon, respectively. Phylogenetic analysis of these MLO family proteins was performed to find candidate MLO proteins conferring PM susceptibility. In the resulting tree, MELO3C012438P1 and Cla020573 were clustered in the same clade as CsMLO1, AtMLO2, PsMLO1 and SlMLO1 (Supplementary Figure S6A). Sequence alignment showed that CsMLO1, MELO3C012438P1 and Cla020573 were highly conserved, especially in the seven predicted transmembrane domains (TM1–TM7), the CaMBD region and domains I and II (Supplementary Figure S6B). Therefore, MELO3C012438P1 and Cla020573 might be negative regulators mediating PM resistance in melon and watermelon, respectively. Blast analysis of MELO3C012438P1 showed that it was an allele of CmMLO2, which was cloned via the RACE method from melon and might play role in the pathogenesis of PM (Cheng et al., 2013).

### qRT-PCR of Signaling and Resistance Pathway Genes

**FIGURE 6:**
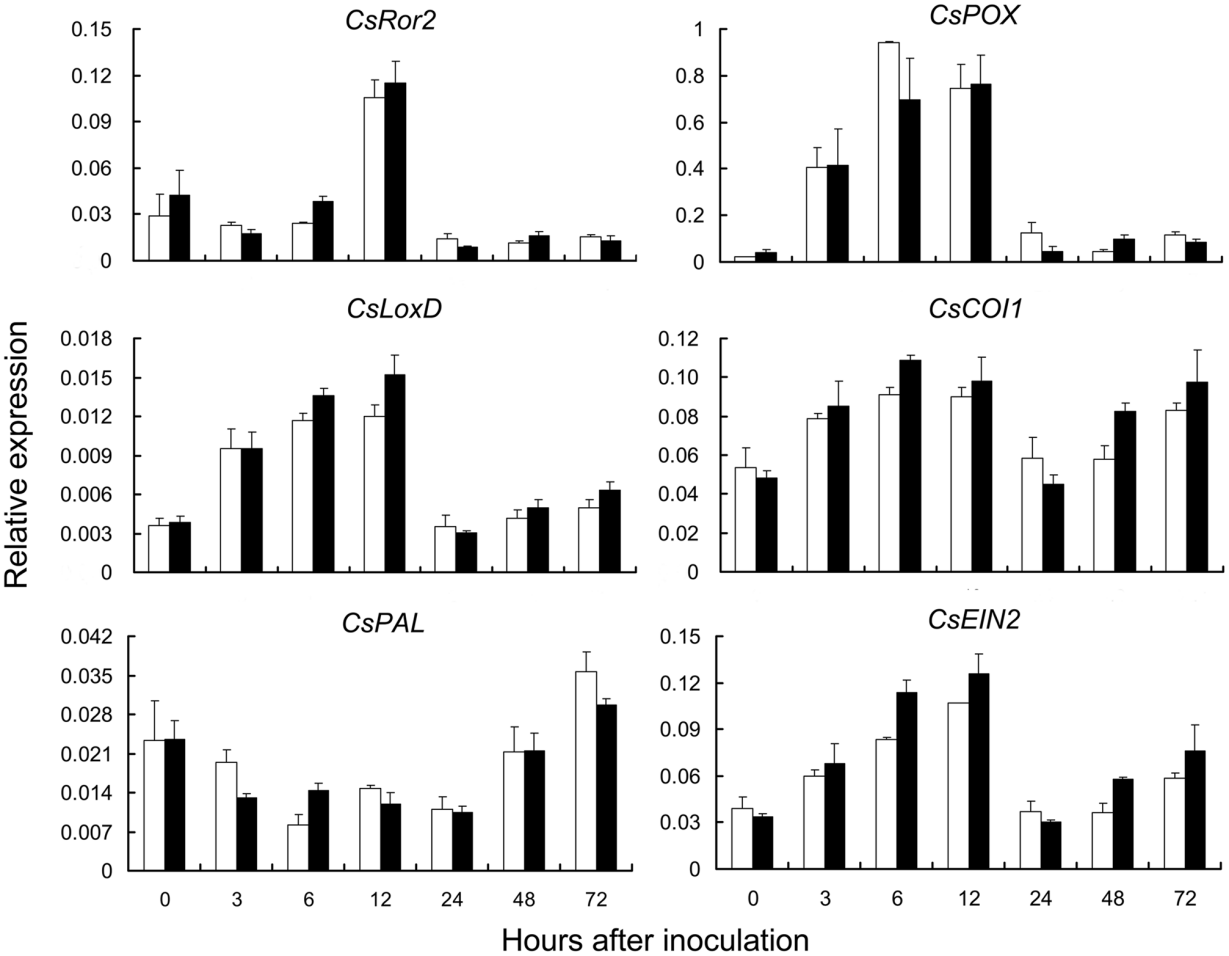
**Expression patterns of plant defense-response related genes in resistant S1003 and susceptible NIL(*Pm5.1*) plants after PM inoculation**. *CsRor2* is an ortholog of barley *Ror2*, which is required for PM resistance. *CsPOX* is a peroxidase gene. *CsLoxD* and *CsCOI1* are JA synthesis-related genes. *CsPAL* is an SA synthesis-related gene. *CsEIN2* is an ethylene signaling pathway receptor gene. The cucumber *CsActin* gene was used as a reference control. Open bars, resistant S1003; solid bars, susceptible NIL(*Pm5.1*). Values are mean ± SE (*n* = 3). No genes showed a significant difference between S1003 and NIL(*Pm5.1*) at any time point after inoculation.

Plant defense responses include the activation of pathways dependent on SA and JA/ethylene signal molecules (Qiu et al., 2007; Du et al., 2009). To investigate the pathway involved in *pm5.1*-mediated resistance, the expression patterns of genes that affect PM resistance in other pathosystems and defense-responsive genes that function in SA- and JA/ethylene-dependent pathways were examined (Freialdenhoven et al., 1996; Li et al., 2007; Qiu et al., 2007). There was no significant difference between the S1003 and NIL(*Pm5.1*) plants in the transcript levels of the SA synthesis-related gene *CsPAL* (phenylalanine ammonia-lyase), the JA synthesis-related genes *CsLOX* (lipoxygenase) and *CsCOI1* (coronatine-insensitive 1) or the ethylene signaling pathway receptor gene *CsEIN2* (ethylene insensitive 2) (Xie et al., 1998; Jun et al., 2004; Qiu et al., 2007) after PM inoculation (**Figure 6**). These results suggested that the resistance difference between S1003 and NIL(*Pm5.1*) had no correlation with expression levels of these genes in SA- or JA/ethylene-dependent pathways. However, the transcripts of these genes increased or decreased within 12 h after PM inoculation and subsequently returned to normal levels. We need more evidences to find the crosstalk of *pm5.1*-mediated resistance with these pathways.

In addition, *CsRor2* is an ortholog of barley *Ror2*, a syntaxin gene required for PM resistance in barley (Freialdenhoven et al., 1996), and *CsPOX* is a peroxidase gene. *CsRor2* and *CsPOX* accumulated after inoculation and then decreased rapidly after 12 hpi, but the transcript levels of the two genes showed no significant difference between S1003 and NIL(*Pm5.1*), (**Figure 6**), suggesting that the difference of PM resistance between S1003 and NIL(*Pm5.1*) had no correlation with expression levels of these two genes.

### Characterization of DEGs between S1003 and NIL(*Pm5.1*)

To further elucidate the resistance mechanism mediated by *pm5.1*, transcriptome analysis was performed to identify DEGs between S1003 and NIL(*Pm5.1*). *mlo*-mediated resistance to PM functions in the early infection stage when germinating fungal spores enter epidermal host cells (Jørgensen, 1992; Büschges et al., 1997). In successful infection of barley PM *Bgh*, haustoria develop within the host cell and are visible during 12–14 hpi, which is similar in all PMs. Therefore, considering this infection stage together with the fact that the transcript levels of genes showed large changes at 12 hpi (**Figures 3D** and **6**), the leaves of the two NILs were sampled at 12 hpi and before inoculation for DGE profile analysis.

**FIGURE 7:**
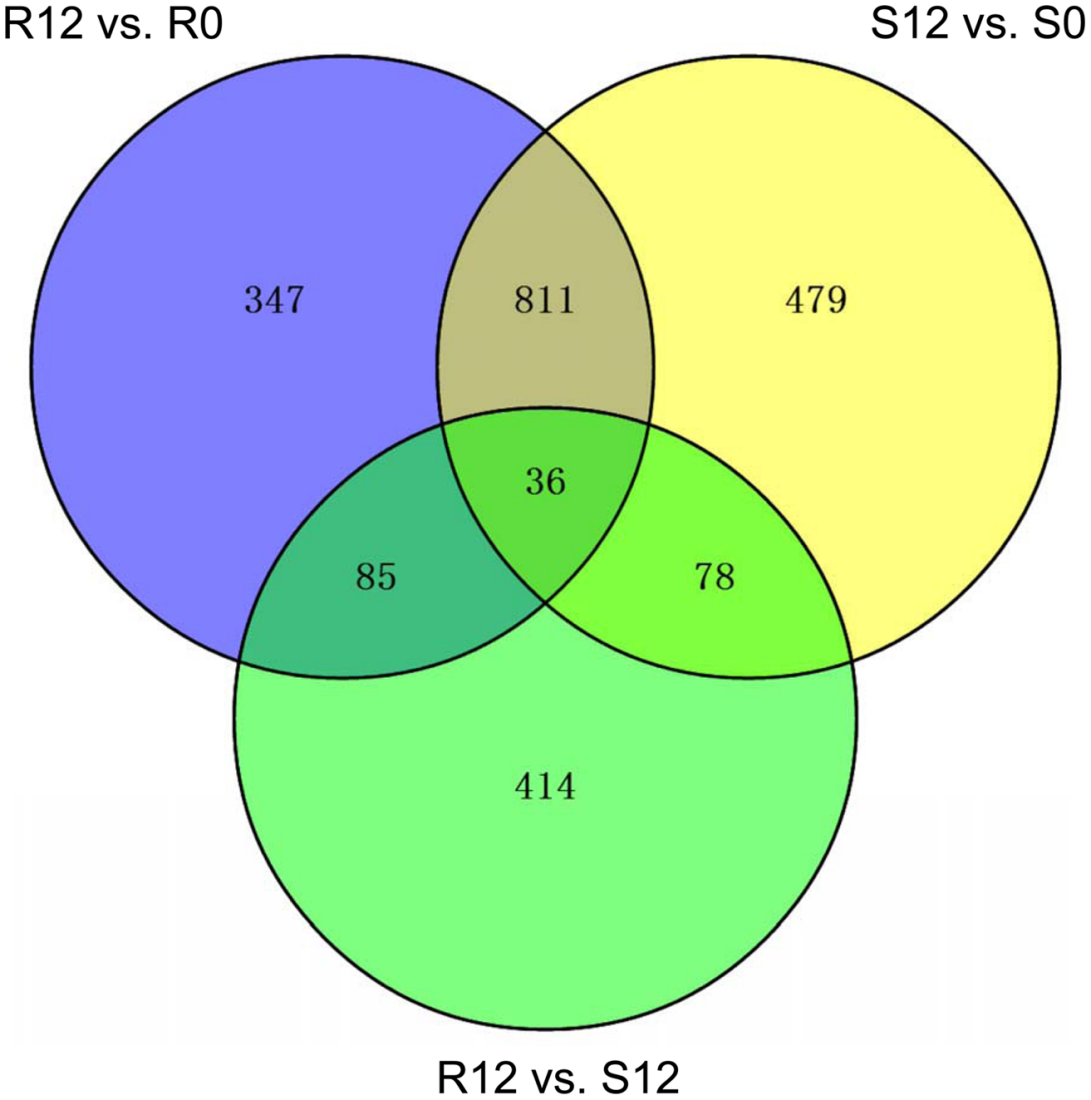
**Venn diagrams of differentially expressed genes (DEGs)**. The DEG sets (R12 vs. R0, S12 vs. S0 and R12 vs. S12) were analyzed using the Venn method. The numbers marked in the diagram indicate the number of genes significantly differentially expressed among the three DEG sets (log_2_-fold change > 1 or < –1 and *P*-value < 0.05). Eighty-five DEGs were defined as resistance-specific expressed genes (RSGs) in S1003, and 36 DEGs were defined as constitutively expressed genes (CEGs) in S1003 and NIL(*Pm5.1*).

Three DEG groups were analyzed, R12 vs. R0, S12 vs. S0 and R12 vs. S12. There were 1279, 1404, and 613 DEGs in the three groups, respectively (**Figure 7**). Venn analysis was used to screen for specific genes correlated with PM resistance or susceptibility (**Figure 7**). A total of 121 DEGs were common to the R12 vs. R0 and R12 vs. S12 DEG groups. Of those, 36 DEGs were common to the S12 vs. S0 DEG group and were considered to be constitutively expressed genes (CEGs) in S1003 and NIL(*Pm5.1*) (**Figure 7**; Supplementary Table S5). The remaining 85 DEGs were defined as resistance-specific expressed genes (RSGs) in S1003 (**Figure 7**; Supplementary Table S5). GO analysis demonstrated that the RSGs were mainly distributed in the ion transport, phenylpropanoid metabolism (GO-biological process), cytochrome complex, plant-type cell wall (GO-cellular component), glycosyltransferase activity, and transporter activity (GO-molecular function) categories (Supplementary Figure S7; Supplementary Table S6).

**FIGURE 8:**
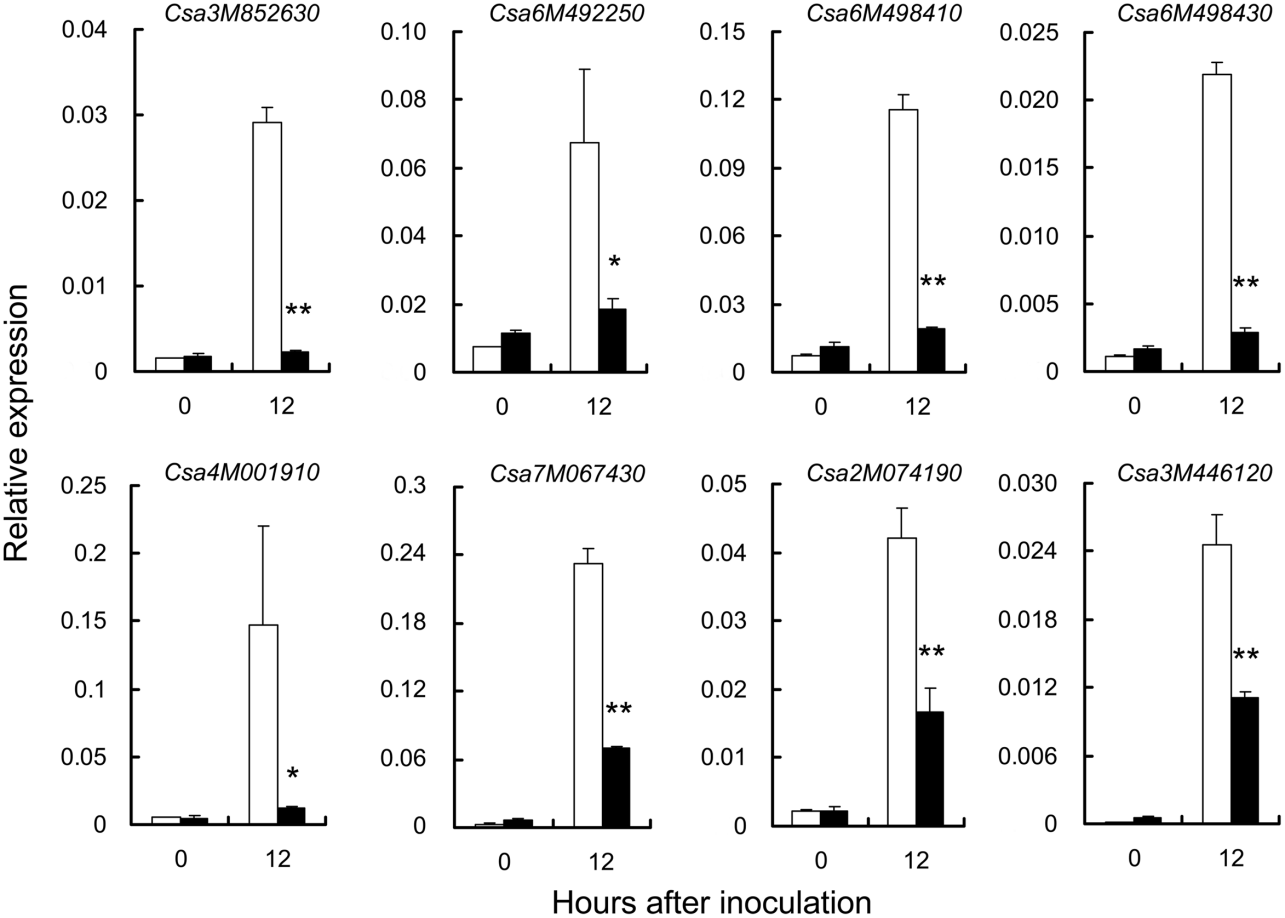
**Verification of differentially expressed genes (DEGs) by qRT-PCR**. DEGs were randomly chosen from the RSG and CEG sets in **Figure 7**. The cucumber *CsActin* gene was used to quantify the relative transcript levels. Open bars, resistant S1003; solid bars, susceptible NIL(*Pm5.1*). Values are mean ± SE (*n* = 3) (^∗^ and ^∗∗^ indicate significant differences between S1003 and NIL(*Pm5.1*) at *P* = 0.01 and 0.05, respectively).

To explore the genes related to PM resistance in S1003, several RSGs and CEGs associated with the main GO terms and correlated with defense responses (Supplementary Table S6) were chosen randomly. The expression of these RSGs and CEGs was confirmed by qRT-PCR (**Figure 8**). Notably, five genes (*Csa3M852630.1*, *Csa6M492250.1*, *Csa6M498410.1*, and *Csa6M498430.1* in the RSGs; *Csa3M446120.1* in the CEGs) were related to “defense response by cell wall thickening” (GO:0052482), and “defense response by callose deposition” (GO:0052542; Supplementary Table S6). In barley, loss of function of the *MLO* gene conferred PM resistance that stopped infection through cell wall thickenings called papillae, which formed beneath infection sites during cell wall penetration (Jørgensen, 1992; Büschges et al., 1997). Same circumstances were occurred in *Arabidopsis* and tomato (Nishimura et al., 2003; Bai et al., 2005). Callose is a major component of papillae and is deposited in papillae during the early stage of host-pathogen interaction (Nishimura et al., 2003; Romero et al., 2008; Lionetti and Métraux, 2014; Voigt, 2014). Therefore, the results indicated that plant cell wall thickening might play an important role in PM resistance mediated by *pm5.1* in S1003. Two ABC transporter genes (*Csa2M074190.1* in the RSGs, *Csa3M446120.1* in the CEGs) were both up-regulated post inoculation but more significantly in S1003 than in NIL(*Pm5.1*) (**Figure 8**). *Csa3M446120.1* is a cucumber ortholog of *Arabidopsis PEN3*, and it might function in plant cell wall thickening as described above. Previous results showed that *PEN3* encodes a pleiotropic drug-resistance protein required for *mlo* resistance in *Arabidopsis* (Consonni et al., 2006). Therefore, the ABC transporter might also be closely correlated with the *pm5.1*-mediated PM resistance in S1003.

## Discussion

Powdery mildew is a common disease of higher plant species and causes substantial economic losses. Widespread use of protective fungicides to control the disease is prevalent because race-specific *R* resistance is easily lost. Therefore, durable and broad-spectrum resistance genes are urgently required for breeding resistant varieties to ensure crop production. Mutant or naturally defective alleles of the *MLO* gene confer durable and broad-spectrum resistance to PM (Büschges et al., 1997; Piffanelli et al., 2004; Consonni et al., 2006; Bai et al., 2008; Humphry et al., 2011). Recently, TALEN (transcription activator-like effector nuclease)-induced mutation of all three *TaMLO* homologs in one hexaploid bread wheat plant conferred heritable broad-spectrum resistance to PM (Wang et al., 2014).

The *MLO* gene family contains different members in diverse plant species. The sequenced rice, wheat, maize and *Arabidopsis* genomes encode 11, 7, 9 and 15 predicted MLO proteins, respectively, and bread wheat contains three close homologs of barley *MLO*, which most likely represent orthologs from the A, B, and D genomes (Devoto et al., 2003; Collins et al., 2007; Konishi et al., 2010). However, only members characterized by the presence of a clade-specific peptide motif, the D/E-F-S/T-F tetrapeptide, at the highly polymorphic C-terminus are correlated with PM resistance (Panstruga, 2005b; Humphry et al., 2011). In barley, loss of function of a single *MLO* gene results in full PM resistance, while unequal genetic redundancy exists between three phylogenetically closely related *MLO* co-orthologs (*AtMLO2*, *AtMLO6*, and *AtMLO12*) in *Arabidopsis*. Although a similar number of *MLO* family genes were predicted in cucumber to *Arabidopsis*, only *CsMLO1* was clustered in the same clade as *AtMLO2*, *AtMLO6* and *AtMLO12* (**Figure 2**; Supplementary Figure S6), indicating loss of function of only one *MLO* gene, *CsMLO1*, can confer full resistance to PM in cucumber. The case may be the same in melon and watermelon (Supplementary Figure S6). However, full PM resistance in cucumber may require other gene functions. The quantitatively inherited nature of PM resistance in cucumber supports this (Sakata et al., 2006; Liu et al., 2008a,b; Zhang et al., 2011; Fukino et al., 2013; He et al., 2013; Nie et al., 2015). In barley, *Ror1* and *Ror2* are required for *mlo* resistance (Freialdenhoven et al., 1996; Collins et al., 2003). *mlo* resistance in *Arabidopsis* requires a syntaxin, glycosyl hydrolase and ABC transporter, encoded by *PEN1*, *PEN2* and *PEN3*, respectively (Consonni et al., 2006). The orthologs of these genes in cucumber might play important role in PM resistance together with *Csmlo1*.

*CsMLO1* complemented the loss of function of *AtMLO2* and *AtMLO12*, indicating that cucumber has a resistance mechanism similar to *mlo2*-based resistance in *Arabidopsis*. Likewise, barley *mlo* mutants terminate essentially all wheat PM entry attempts and over-expression of the barley *MLO* gene or a wheat homolog in cultivated bread wheat resulted in PM super susceptibility (Elliott et al., 2002). All these findings show that the function of MLO is evolutionarily conserved, and loss of MLO function conferring PM resistance is ancient. Previous findings imply that a common host cell entry mechanism evolved once in PM fungi at least 200 million years ago, suggesting that within the Erysiphales (PMs) the ability to cause disease has been a stable trait throughout phylogenesis (Consonni et al., 2006). Since monocots are believed to have diverged from dicots approximately 100–270 million years ago, *MLO*-like genes must have already existed in their common progenitor (Wolfe et al., 1989; Devoto et al., 2003). Therefore, all of these findings together indicate that the loss of MLO function conferring PM resistance might predate the divergence of monocots and dicots.

In the present study, cucumber inbred lines originating from various geographical regions of the world (Supplementary Table S1) were used to analyze the PM resistance phenotype and the allele sequences of *CsMLO1*. The susceptible haplotypes were conserved within the cultivated and wild cucumber lines, but three independent haplotypes were present among the resistant domesticated and wild cucumber lines (*pm5.1*-mediated or unknown). The resistant haplotypes included a large DNA insertion disrupting the gene, SNP exchange in the splice site resulting in mis-splicing, and SNP insertion causing a premature stop codon (**Table 1**). These results strongly indicate that *CsMLO1* is identical to *Pm5.1* and that CsMLO1 is a negative regulator of PM resistance in cucumber. Interestingly, 1449-bp and 1451-bp DNA fragment insertions occurred in two independent mutation events, haplotype A and cucumber line CGN20854, respectively (Supplementary Figure S4). Analysis of the insertion sequences showed that integration of a *Copia*-like LTR-retrotransposon caused these two insertion mutations of *CsMLO1*. Because PM makes use of the function of MLO for pathogenesis, this might have been an effective way for cucumber to resist infection of the PM pathogen over the evolutionary history of the host–pathogen battle. However, the insertion mutation in CGN20854 did not change the expression of the normal transcript and therefore the susceptible phenotype. Moreover, haplotype A was prevalent in the resistant cucumber lines, for two possible reasons. First, this mutation event could occur relatively easily. Second, this mutation occurred early in cucumber evolution and domestication, and then spread worldwide because it conferred resistance to PM. This hypothesis should be verified by sequencing more cucumber lines and by studying the history of cucumber domestication (Qi et al., 2013). However, the causative polymorphisms resulting from these three independent natural mutations will be useful in marker-assisted selection for molecular breeding of PM-resistant cucumber varieties in the near future.

Within the 28 cucumber inbred lines, S94 and 9930 were exceptions that had resistant haplotypes but exhibited the susceptible phenotype. This phenomenon could be explained as described above. The loss of function of *CsMLO1* may be necessary but not sufficient for PM resistance in cucumber because other gene functions are needed, just like *Ror1* and *Ror2* are required for *mlo* resistance in barley (Freialdenhoven et al., 1996; Collins et al., 2003). Genetic analysis and gene mapping using PM susceptible and resistant cucumber lines in the *Csmlo1* resistant background should uncover the genes required for *mlo* resistance in cucumber. Indeed, QTL analysis for PM resistance was performed using S94 and S06, which are in the *Csmlo1* background (Liu et al., 2008a,b), and the QTLs obtained may be factors required for *mlo* resistance in cucumber.

The defense response genes *PAL*, *LOX*, *COI1* and *EIN2* are known to function in SA- and JA/ethylene-dependent pathways. In this study, the transcript levels of their homologs in cucumber did not show any significant difference between the resistant S1003 and susceptible NIL(*Pm5.1*) lines at the early stage of PM infection. In *Arabidopsis*, *mlo* resistance does not involve the signaling molecules ethylene, JA or SA (Consonni et al., 2006). Therefore, the results indicated that the same case might occur in *mlo*-mediated PM resistance in cucumber. However, it needs more works to be clarified in near future. Additionally, transcriptome analysis was performed to identify genes and the possible mechanism involved in *pm5.1*-mediated PM resistance in cucumber. GO analysis of the RSGs and qRT-PCR showed that *pm5.1*-mediated resistance in S1003 was correlated with plant cell wall thickening (Supplementary Figure S7; **Figure 8**). Therefore, the expression changes of the RSGs after PM inoculation might provide a physical and/or chemical barrier to resist pathogen entry at the plant cell wall. These results provide a solid foundation for further studies on the mechanism of PM resistance in cucumber.

In this study, recessively inherited *pm5.1*-mediated PM resistance was shown to be caused by loss of Cs*MLO1* function. The chain of evidence includes phylogenetic analysis, a complementation test of *CsMLO1* in *Arabidopsis*, as well as independent natural mutation events of *CsMLO1* in PM-resistant lines (*pm5.1*-mediated or unknown) of different geographical origins. The cucumber line S1003 has been highly resistant to PM for several decades in China; therefore, *pm5.1* provides effective and durable disease resistance, which is very valuable for cucumber breeding. The *pm5.1* allele is the first example of a major locus conferring for PM resistance that was isolated using a positional cloning approach in cucurbit crops and is one of few examples, besides barley *mlo*, *Arabidopsis AtMLO2*, tomato *SlMLO1*, and pea *PsMLO1*, of a cloned natural or induced *mlo* mutant gene (Büschges et al., 1997; Piffanelli et al., 2004; Consonni et al., 2006; Bai et al., 2008; Humphry et al., 2011). These findings, together with the conclusion that loss of MLO function conferring PM resistance might predate the divergence of monocots and dicots, indicate that broad-spectrum *mlo*-mediated PM resistance might be prevalent in higher plants, including all the cucurbit crops. Implicit in this is that the engineering of broad spectrum and durable *mlo*-mediated PM resistance might be feasible in any higher plant species. This conclusion needs to be supported by cloning and verification of more *mlo*-based PM resistance genes in more plant species. However, the data presented here show that loss of *CsMLO1* function confers durable PM resistance in cucumber, adding another example of *mlo*-mediated resistance in higher plants. This will facilitate the molecular breeding of PM resistance in cucumber, and other cucurbit crops.

## Author Contributions

JN, YW, HL, JSP, and RC designed the study and interpreted the data. JN, YW, HH, CG, WZ, and JP performed the experiments. JN, YW, DL, HL, and JSP analyzed data. JN, JSP, and RC wrote the paper. All authors discussed the results and commented on the manuscript.

## Conflict of Interest Statement

The authors declare that the research was conducted in the absence of any commercial or financial relationships that could be construed as a potential conflict of interest.

## Footnotes

1 https://www.arabidopsis.org/servlets/TairObject?type=germplasm\&id=6530492878

2 http://www.softberry.com/

3 http://bioinformatics.psb.ugent.be/webtools/plantcare/html/

4 http://dnaman.software.informer.com/6.0/

5 http://www.icugi.org/cgi-bin/ICuGI/genome/home.cgi?organism=cucumber\&ver=2\&cultivar=Chinese-long

6 http://topcons.cbr.su.se/

7 http://www.cbs.dtu.dk/services/TMHMM-2.0/

8 http://www.ncbi.nlm.nih.gov/pubmed/25260700

9 http://www.genomebiology.com/2010/11/10/r106

10 http://www.icugi.org/cgi-bin/ICuGI/genome/home.cgi?ver=2\&organism=cucumber\&cultivar=Chinese-long

11 http://www.icugi.org/cgi-bin/ICuGI/genome/index.cgi?organism=cucumber

12 http://bioinfogp.cnb.csic.es/tools/venny/index.html

13 https://www.arabidopsis.org/index.jsp
