# Supplementary material for: Loss-of-Function Mutations in CsMLO1 Confer Durable Powdery Mildew Resistance in Cucumber (Cucumis sativus L.)
Source: Front Plant Sci. 2015 Dec 22;6:1155. doi: 10.3389/fpls.2015.01155 (PMC4687411; doi:10.3389/fpls.2015.01155)
Supplement: Supplementary file 1 [file Data_Sheet_1.PDF]

Frontiers in Plant Science

***Supplementary Material*:**

Loss-of-function mutations in *CsMLO1* confer durable powdery mildew resistance in cucumber (*Cucumis sativus* L.)

Jingtao Nie [1], Yunli Wang [1], Huanle He [1], Chunli Guo [1], Wenying Zhu [1], Jian Pan [1], Dandan Li [2], Hongli Lian [1], Junsong Pan [1*], Run Cai [1*]

* Correspondence:

Junsong Pan, Run Cai

E-mail: jspan71@sjtu.edu.cn; cairun@sjtu.edu.cn

Supplementary Table 1. The cucumber inbred lines used in this study.

Code	Cucumber material	Ecotype	Origin	Phenotype*	Haplotype[#]
1	S1003	East Asian type	China	R	A
2	S05	Eurasian type	Israel	S	-
3	S1001	East Asian type	China	S	-
4	S02	Eurasian type	Israel	R	A
5	S06	Eurasian type	Israel	R	A
6	R077	East Asian type	China	R	B
7	R078	East Asian type	China	R	B
8	R079	East Asian type	China	R	B
9	PI197088-R	Indian wild type	India	R	C
10	PI197088-S	Indian wild type	India	S	-
11	Aonaga Suyo Kyuri (CGN20854)	East Asian type	Japan	S	-
12	WI2757	Eurasian type	United States	R	A
13	True Lemon	Eurasian type	United States	S	-
14	Gy14	Eurasian type	United States	R	A
15	316	Xishuangbanna type	China	S	-
16	2003502	Eurasian type	China	S	-
17	Hardwickii	Indian wild type	India	S	-
18	S52	East Asian type	China	S	-
19	Straight.Eight	Eurasian type	United States	S	-
20	*gl*	East Asian type	China	S	-
21	H34	Eurasian type	China	R	A
22	S77	Eurasian type	China	R	A
23	WI1983G	Eurasian type	United States	R	A
24	422	Eurasian type	China	R	A
25	115	East Asian type	China	R	B
26	106	East Asian type	China	R	A
27	S94	East Asian type	China	S	A
28	9930	East Asian type	China	S	B

* The phenotype in *Podosphaera xanthii* inoculation tests: R, resistant; S, susceptible; [#] Haplotypes are as described in Table 1; - susceptible lines in which the amino acid sequences of their *CsMLO1* alleles were identical to those of S05 or S52 (Figure S3B)

Supplementary Table 2. The primers used in this study.

Analysis	Primer name	Sequence (5′-3′)	Size(bp) S1003/S05
CDS region of *CsMLO1* for subcellular localization and genetic transformation	SUB-F	GACTAGTATGGCTGAATGTGGAACAGAG	1739
	SUB-R	CGAGCTCTCATTTGGCAAATGAGAAGTCT	
Sequencing of the *CsMLO1* gene in different cucumber inbred lines	N7-F	TTCATTTCCTCTCCTTATTGGT	4484/5933
	N14-R	CATCGTTTTGTTTTAGATTGGAA	
	N7-R	CTGGCTTGTGCCTTTTCTC	-
	N8-F	TTTTTATCTTTTGTTGAAGTTGTG	-
	N9-F	GAATTTCATTTTTCTGCTCAGTA	-
	N10-F	TACTGAGCCTTTTGTGATGACTA	-
	N11-F	TTTGTGTCTCCTGATTCACTTCT	-
	N12-F	TTCCTTAGTTCCTTTCAATCTCT	-
	N13-F	AGACCATTTTTCAATCCAACTTT	-
	N14-F	CACCACTCAGCCAAGAAGAAC	-
Sequencing of the predicted promoter of *CsMLO1*	N2-F	TCCCTCCACTGTCATTTTTTT	2820
	N6-R	TCCAGTGAGGTGAATGACATG	
	N2-R	TCTACTCATCGGAAGAACATTTT	-
	N3-F	CATCTCCCACCTATACCTAATCA	-
	N4-F	ACGAGAAAGAAGGCCGATAT	-
	N5-F	ATCTTAACTATACCTATACCCCCC	-
	N6-F	GAACCCCTTGCTTTGACTTAG	-
Sequencing of the cDNA of *CsMLO1*	MLO-F	GCATCTACTTGTTTCATCCTTCTC	2086
	MLO-R	ACATTGGTGTGGAGGAGGAG	
PCR test of transgenic *Arabidopsis* plants	GM-F	GCAGAAGAACGGCATCAAGG	390
	GM-R	CTTTTCTCCAGCCACTTTCCAG	
Semi-quantitative RT-PCR for spatial expression patterns of *CsMLO1*	RT5-F	TTTTCATCTTCGTATTGGCTGTC	112
	RT5-R	ATTGTCTTGGTTTCATCCTCCC	
CsMLO1 for qRT-PCR	DRT2-F	CTGGGAGGATGAAACCAAGAC	153
	DRT2-R	AACTGTCTGAAGAAACAAACAATCC	
Semi-quantitative RT-PCR for determining changes in transcripts	MRT-F	GCATCTACTTGTTTCATCCTTCTC	1915
	MRT-R	AGATGTAGTATTTGCTGCTGCG	
qRT-PCR for determining the transcript level of CGN20854	P1	GCCAACTCGCTTTCGTGTC	133
	P2	GCCTTCCATTTGCTCATCTTT	
	P3	TTTCCACAAATACATTAGCAGATCTC	115
	P4	CCACCCATTTGTATTGGTTAGGA	
	P3	TTTCCACAAATACATTAGCAGATCTC	144
	P5	GAGATGAAAGGCAGCCATAGAT	
	P6	TTCTCTTCATCCTAACCAATACAAAT	152
	P7	CTTCACAACATGACCCCTTTCT	

CsRor2 for qRT-PCR	CsRor2-F<br>CsRor2-R	TACACTGTTACTGGTGAAAATCCG<br>AGATTCTGCCTCTGCCTTGTTC	115
CsPOX for qRT-PCR	CsPOX-F<br>CsPOX-R	AATGAAACCGACCTTGTTGC<br>TTGGACAAACGACCACTGAA	83
CsLoxD for qRT-PCR	CsLoxD-F<br>CsLoxD-R	ATAGATTTTCTTGGCTGAGGGAC<br>TTAGGGTCAAGTTTGCTACGAAT	115
CsEIN2 for qRT-PCR	CsEIN2-F<br>CsEIN2-R	ATTGTAAGTTTTGGTGTATGGTGC<br>GGATCGATAATACCCTGAAGACG	122
CsCOI1 for qRT-PCR	CsCOI1-F<br>CsCOI1-R	CAGAGATGCCCCAACTTAGAAAT<br>GACCTTCCTCGTCTTCTAATCCC	148
CsPAL for qRT-PCR	CsPAL-F<br>CsPAL-R	ACTCCATGTTTGCCTCTTCG<br>AAGCCTCCTTTGCATCAAGA	142
Csa3M852630.1 for qRT-PCR	3M852630-F<br>3M852630-R	TGTTAGAGATGACGGACAACTTTC<br>ATTGTTACTGTTGTAGTGTCAGATCC	105
Csa6M492250.1 for qRT-PCR	6M492250-F<br>6M492250-R	AAGGAAGAAGGAAGGAACACGAT<br>TCGGATCCAGCCGTTAGTAATAT	122
Csa6M498410.1 for qRT-PCR	6M498410-F<br>6M498410-R	AACTTCCTTTCCCTCAAATCAAT<br>TGGGTCCGACAAATACAAGAAAT	114
*Csa6M498430.1*for qRT-PCR	6M498430-F<br>6M498430-R	TTGGATCTTGTTCGACTTCGC<br>TCGGGACTATAAATACTAAAGAACGC	96
Csa3M446120.1 for qRT-PCR	3M446120-F<br>3M446120-R	GAGCAGCCTGATTACTGACTACAC<br>TCTTTTCCTTTGTCCACCCGAT	112
Csa2M074190.1 for qRT-PCR	2M074190-F<br>2M074190-R	CAGCGGCTTTTATCCAGGTA<br>TCCTCTCAACCACCTCTCCG	147
Csa7M067430.1 for qRT-PCR	7M067430-F<br>7M067430-R	ACATGAAATGGTTCGGGATAAT<br>ATCACTGAACTTGTTGGTTGCTG	203
Csa4M001910.1 for qRT-PCR	4M001910-F<br>4M001910-R	GTTCTCCCATTCTGGCTTCAC<br>ACCTTTATTTCTGCCGCTGTC	99
Cucumber *CsActin* gene for RT-PCR and qRT-PCR	Actin-F<br>Actin-R	TCGTGCTGGATTCTGGTG<br>GGCAGTGGTGGTGAACAT	161

- Primers used for sequencing

Supplementary Table 3. *Cis*-acting regulatory elements in the promoter sequences of *CsMLO1* alleles in S05, S1003, and S1001. Positions are based on the promoter sequence of the *CsMLO1* allele of S1003. Gray shading indicates elements not found in S05.

Element	Position(bp)	Matrix score.	Sequence	Function
Box-W1	-1270	6	TTGACC	Fungal elicitor responsive element
	-225	6	TTGACC	
	-849	6	TTGACC	
TC-rich repeats	-1031	9	ATTTTCTTCA	*Cis*-acting element involved in defense and stress responsiveness
	-430	9	ATTTTCTTCA	
	-666	9	ATTTTCTTCA	
	-314	9	ATTTTCTTCA	
	-782	10	GTTTTCTTCA	
	-614	9	ATTTTCTTCA	

Supplementary Table 4. Allelism test of the locus conferring powdery mildew resistance.

Parents and combination	Population	Total	Resistant	Susceptible
S1003	-	30	30	0
R077	-	30	30	0
PI197088-R	-	30	30	0
422*	-	30	30	0
S05	-	30	0	30
S1003×R077	F_1	30	30	0
	F_2	52	52	0
S05×R077	F_1	15	0	15
PI197088-S×PI197088-R	F_1	14	0	14
422×PI197088-R	F_1	10	10	0
	F_2	183	183	0

- indicates the parent cucumber inbred lines; * the sequence of the *CsMLO1* allele in line 422 was identical to that of S1003.

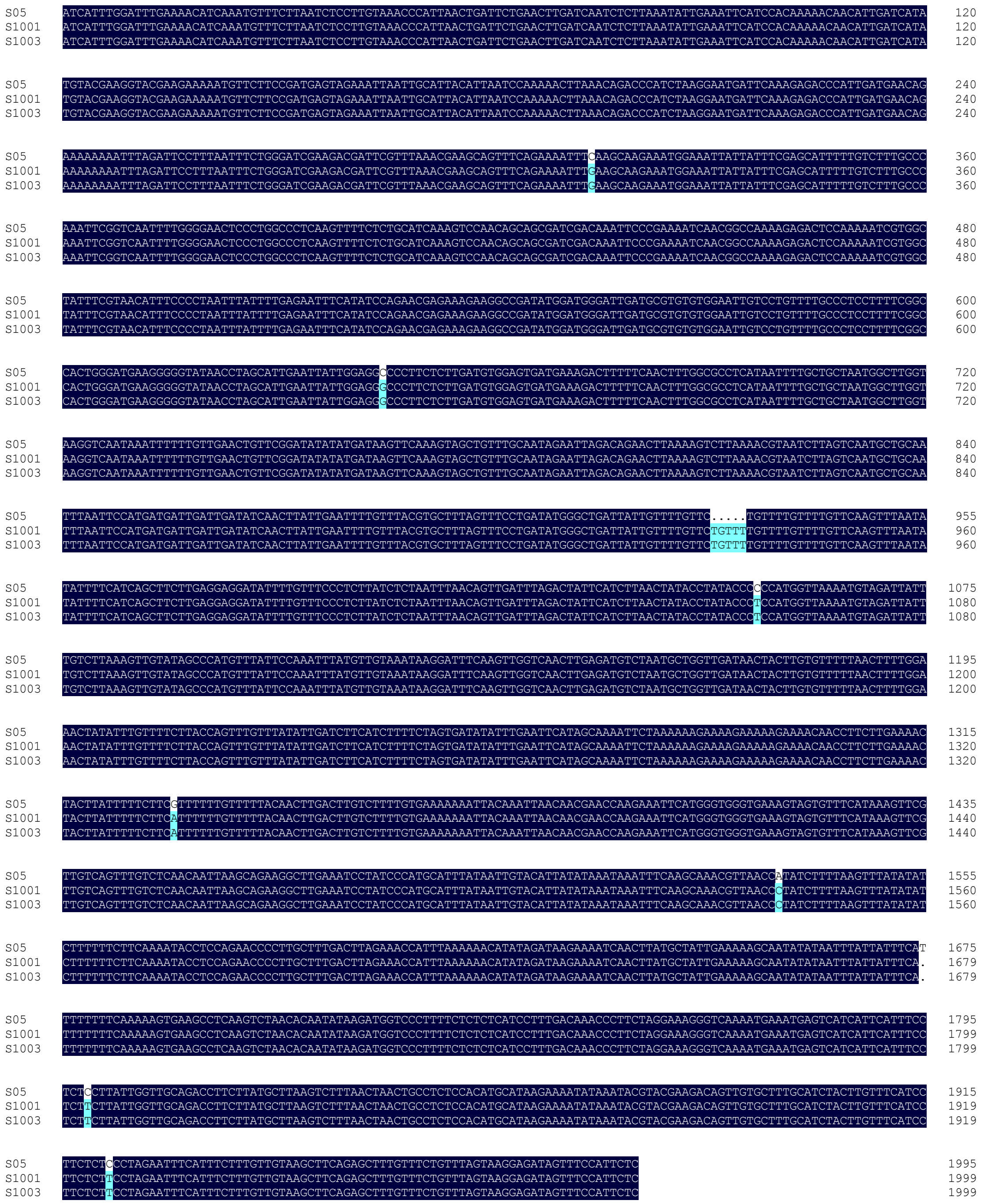

Supplementary Figure 1. Comparison of the putative promoter sequences of *CsMLO1* alleles in S05, S1003, and S1001.

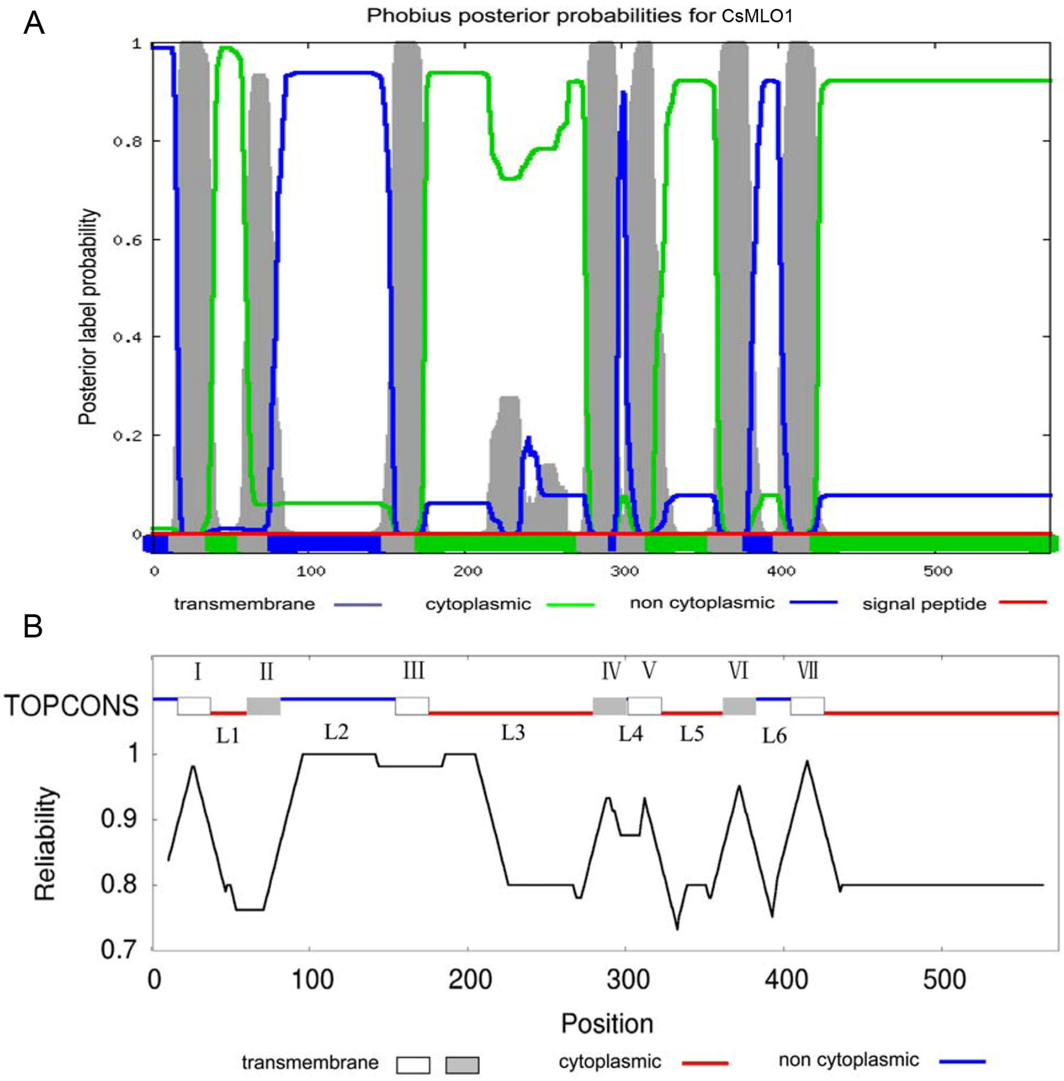

Supplementary Figure 2. The predicted transmembrane topology of the CsMLO1 protein analyzed using the online tools Phobius (http://phobius.sbc.su.se/) (A) and TOPCONS (http://topcons.cbr.su.se/) (B). Cytoplasmic and non-cytoplasmic represent the intracellular and extracellular locations, respectively. Amino acids 1–16 at the N-terminus are extracellular, and amino acids 427–574 at the C-terminus are intracellular. I–VII denote the positions of the seven transmembrane helixes formed by the hydrophobic segments; L1–L6 represent the loop rings.

A

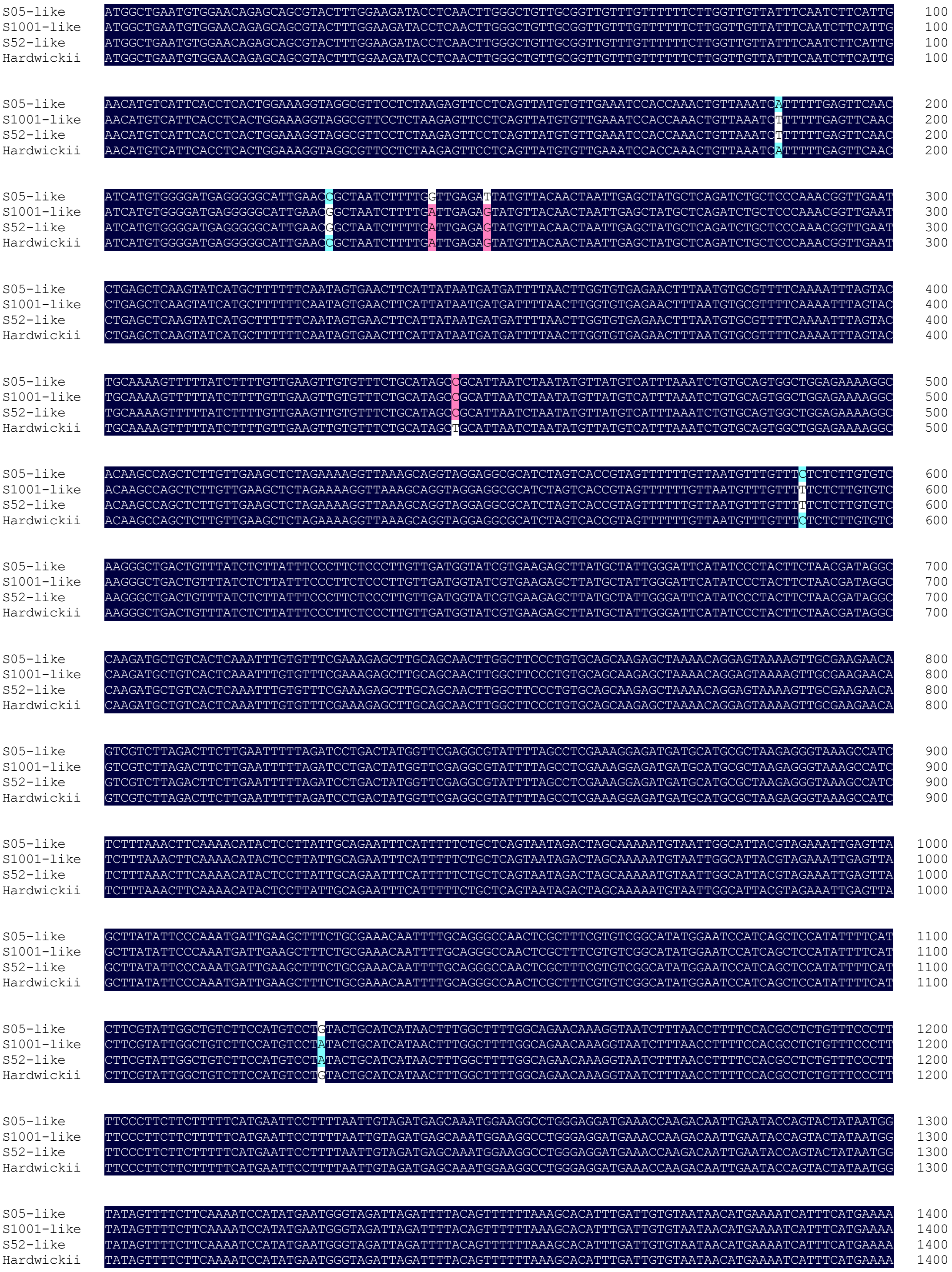

S05-like
S1001-like
S52-like
Hardwickii
100
200
300
400
500
600
700
800
900
1000
1100
1200
1300
1400

```
S05-like     GCTCTTTTAATAACCTATAAATTTTTACTCAAATCACTACTTTTCATACATGCCTCAAGTACTGAGCCTTTTGTGATGACTAGTTGATAGAATCTCATAC   1500
S1001-like   GCTCTTTTAATAACCTATAAATTTTTACTCAAATCACTACTTTTCATACATGCCTCAAGTACCGAGCCTTTTGTGATGACTAATTGATAGAATCTCATAC   1500
S52-like     GCTCTTTTAATAACCTATAAATTTTTACTCAAATCACTACTTTTCATACATGCCTCAAGTACCGAGCCTTTTGTGATGACTAATTGATAGAATCTCATAC   1500
Hardwickii   GCTCTTTTAATAACCTATAAATTTTTACTCAAATCACTACTTTTCATACATGCCTCAAGTACTGAGCCTTTTGTGATGACTAGTTGATAGAATCTCATAC   1500

S05-like     AAGTTGCAGATCGTTTTTCAACAGCATGGGACGAAGTCCTTTTTTTATATTTAATATTTTGCTTCCTTTTTTTAGACACATGATGTCTTTTTTGTCATGT   1600
S1001-like   AAGTTGCAGATCGTTTTTCAACAGCATGGGACGAAGTCCTTTTTTTATATTTAATATTTTGCTTCCTTTTTTTAGACACATGATGTC.TTTTTGTCATGT   1599
S52-like     AAGTTGCAGATCGTTTTTCAACAGCATGGGACGAAGTCCTTTTTTTATATTTAATATTTTGCTTCCTTTTTTTAGACACATGATGTC.TTTTTGTCATGT   1599
Hardwickii   AAGTTGCAGATCGTTTTTCAACAGCATGGGACGAAGTCCTTTTTTTATATTTAATATTTTGCTTCCTTTTTTTAGACACATGATGTCTTTTTTGTCATGT   1600

S05-like     TATCAACATCTCTTACTGCAG.ATGCATTCATTAGAAATTCTAAGCAATTTGTAACTTATGAATTGATAATTTCTGTATTGCAGATCCAGCAAGATTTAG   1699
S1001-like   TATCAACATCTCTTACTGCAAAATGCATTCATTAGAAATTCTAAGCAATTTGTAACTTGTGAATTGATAATTTCTGTATTGCAGATCCAGCAAGATTTAG   1699
S52-like     TATCAACATCTCTTACTGCAAAATGCATTCATTAGAAATTCTAAGCAATTTGTAACTTGTGAATTGATAATTTCTGTATTGCAGATCCAGCAAGATTTAG   1699
Hardwickii   TATTAACATCTCTTACTGCAG.ATGCATTCATTAGAAATTCTAAGCAATTTGTAACTTGTGAATTGATAATTTCTGTATTGCAGATCCAGCAAGATTTAG   1699

S05-like     ATTTGCTAGAGATACTACGTTTGGACGCCGACACTTGAGCTTCTGGAGTCGTACACCAATTTCCCTCTGGATTGTGAGTGTTTGTCTCAAAATTAAATCA   1799
S1001-like   ATTTGCTAGAGATACTACGTTTGGACGCCGACACTTGAGCTTCTGGAGTCGTACACCAATTTCCCTCTGGATTGTGAGTGTTTGTCTCAAAATTAAATCA   1799
S52-like     ATTTGCTAGAGATACTACGTTTGGACGCCGACACTTGAGCTTCTGGAGTCGTACACCAATTTCCCTCTGGATTGTGAGTGTTTGTCTCAAAATTAAATCA   1799
Hardwickii   ATTTGCTAGAGATACTACGTTTGGACGCCGACACTTGAGCTTCTGGAGTCGTACACCAATTTCCCTCTGGATTGTGAGTGTTTGTCTCAAAATTAAATCA   1799

S05-like     AGAAGAATTTGCTCATGACTATGAATTTGACAACCTGGGAAGAACTAAAATTATTCACTCTTGCTAGAAACTCTTAGCAAATTATTATAAAGGATGATGC   1899
S1001-like   AGAAGAATTTGCTCATGACTATGAATTT...AACCTGGGAAGAACTAAAATTATTCACTCTTCCTAGACACTCTTAGCAAATTATTATAAAGGATGATGC   1896
S52-like     AGAAGAATTTGCTCATGACTATGAATTT...AACCTGGGAAGAACTAAAATTATTCACTCTTCCTAGACACTCTTAGCAAATTATTATAAAGGATGATGC   1896
Hardwickii   AGAAGAATTTGCTCATGACTATGAATTTGACAACCTGGGAAGAACTAAAATTATTCACTCTTGCTAGAAACTCTTAGCAAATTATTATAAAGGATGATGC   1899

S05-like     TCTTTTGAGTCGTCCATCAAGGTTTTGATAG.AAAAAATCAAAGAGATCTATAGATGAACGTCTTTCACAATGTTCTAATTGCATTTGTGTCTCCTGATT   1998
S1001-like   TCTTTTGAGTCGTCCATCAAGGTTTTGATAGAAAAAAATCAAAGAGATATATAGATGAACGTCTTTCACAATGTTCTAATTGCATTTGTGTCTCCTGATT   1996
S52-like     TCTTTTGAGTCGTCCATCAAGGTTTTGATAGAAAAAAATCAAAGAGATATATAGATGAACGTCTTTCACAATGTTCTAATTGCATTTGTGTCTCCTGATT   1996
Hardwickii   TCTTTTGAGTCGTCCATCAAGGTTTTGATAG.AAAAAATCAAAGAGATCTATAGATGAACGTCTTTCACAATGTTCTAATTGCATTTGTGTCTCCTGATT   1998

S05-like     CACTTCTGTGTACAATCAAATAGTTTTAATTTCCCACTGTTTCTCTTTATGTATTTTATGGTTTTAAATTCTGAAAAAATTTCAGCGATAGTGATGTTAG   2098
S1001-like   CACTTCTGTGTACAATCAAATAGTTTTAATTTCCCACTGTTTCTCTTTATGTATTTTATGGTTTTAAATTCTGAAAAAATTTCAGCGATAGTGATGTTAG   2096
S52-like     CACTTCTGTGTACAATCAAATAGTTTTAATTTCCCACTGTTTCTCTTTATGTATTTTATGGTTTTAAATTCTGAAAAAATTTCAGCGATAGTGATGTTAG   2096
Hardwickii   CACTTCTGTGTACAATCAAATAGTTTTAATTTCCCACTGTTTCTCTTTATGTATTTTATGGTTTTAAATTCTGAAAAAATTTCAGCGATAGTGATGTTAG   2098

S05-like     TTAATTCATATTCTGTTTCACAGGTTTGTTTCTTCAGACAGTTCTTTGGATCAGTTACCAAGGTTGATTACATGACACTGAGACATGGATTCATTGTTGT   2198
S1001-like   TTAATTCATATTCTGTTTCACAGGTTTGTTTCTTCAGACAATTCTTTGGATCAGTTACCAAGGTTGATTACATGACACTGAGACATGGATTCATCGTTGT   2196
S52-like     TTAATTCATATTCTGTTTCACAGGTTTGTTTCTTCAGACAATTCTTTGGATCAATTACCAAGGTTGATTACATGACACTGAGACATGGATTCATCGTTGT   2196
Hardwickii   TTAATTCATATTCTGTTTCACAGGTTTGTTTCTTCAGACAGTTCTTTGGATCAGTTACCAAGGTTGATTACATGACACTGAGACATGGATTCATTGTTGT   2198

S05-like     AAGTAAAACATGTGAAATTGAAATTGCATCAAATCCACTTTTCATATAGTCAAGATTCTGCTCGTCCATTCTTCTTAACTTGAGCCGTTTTACAGGCACA   2298
S1001-like   AAGTAAAACATGTGAAATTGAAATTGCATCAAATCCACTTTTCATATAGTCAAGATTCTGCACGTCCATTCTTCTTAACTTGAGCCGTTTTACAGGCACA   2296
S52-like     AAGTAAAACATGTGAAATTGAAATTGCATCAAATCCACTTTTCATATAGTCAAGATTCTGCACGTCCATTCTTCTTAACTTGAGCCGTTTTACAGGCACA   2296
Hardwickii   AAGTAAAACATGTGAAATTGAAATTGCATCAAATCCACTTTTCATATAGTCAAGATTCTGCACGTCCATTCTTCTTAACTTGAGCCGTTTTACAGGCACA   2298

S05-like     TCTTGCACCCGGAAGTGAAGTAAAATTTGATTTCCACAAATACATTAGCAGATCTCTGGAAGACGACTTTAAAGTTGTTGTGGGGATTAGGTTTGCTTCT   2398
S1001-like   TCTTGCACCCGGAAGTGAAGTAAAATTTGATTTCCACAAATACATTAGCAGATCTCTGGAAGACGACTTTAAAGTTGTTGTGGGGATTAGGTTTGCTTCT   2396
S52-like     TCTTGCACCCGGAAGTGAAGTAAAATTTGATTTCCACAAATACATTAGCAGATCTCTGGAAGACGACTTTAAAGTTGTTGTGGGGATTAGGTTTGCTTCT   2396
Hardwickii   TCTTGCACCCGGAAGTGAAGTAAAATTTGATTTCCACAAATACATTAGCAGATCTCTGGAAGACGACTTTAAAGTTGTTGTGGGGATTAGGTTTGCTTCT   2398

S05-like     TGATTATATAAATAAATTTGATTTCATTTATTGATTTTTATCATAAAATTTTGACCAATTCTCTAATCTCTGCAGTCCCGCAATGTGGCTATTTGCTGTT   2498
S1001-like   TGATTATATAAATAAATTTGATTTCATTTATTGATTTTTATCATAAAATTTTGACCAATTCTCTAATCTCTGCAGTCCCGCAATGTGGCTATTTGCTGTT   2496
S52-like     TGATTATATAAATAAATTTGATTTCATTTATTGATTTTTATCATAAAATTTTGACCAATTCTCTAATCTCTGCAGTCCCGCAATGTGGCTATTTGCTGTT   2496
Hardwickii   TGATTATATAAATAAATTTGATTTCATTTATTGATTTTTATCATAAAATTTTGACCAATTCTCTAATCTCTGCAGTCCCGCAATGTGGCTATTTGCTGTT   2498

S05-like     CTCTTCATCCTAACCAATACAAATGGTAAGCTTACAATCAGTGCATTTCAGAGAAACGTACTTGTCAATTCCTTAGTTCCTTTCAATCTCTGAGTATTTA   2598
S1001-like   CTCTTCATCCTAACCAATACAAATGGTAAGCTTACAATCAGTGCATTTCAGAGAAACGTACTTGTCAATTCCTTAGTTCCTTTCAATCTCTGAGTATTTA   2596
S52-like     CTCTTCATCCTAACCAATACAAATGGTAAGCTTACAATCAGTGCATTTCAGAGAAACGTACTTGTCAATTCCTTAGTTCCTTTCAATCTCTGAGTATTTA   2596
Hardwickii   CTCTTCATCCTAACCAATACAAATGGTAAGCTTACAATCAGTGCATTTCAGAGAAACGTACTTGTCAATTCCTTAGTTCCTTTCAATCTCTGAGTATTTA   2598

S05-like     TTGAGGAATGCTTGTTTTTTGTTCCATATCAGGGTGGTATTCATATCTATGGCTGCCTTTCATCTCCTTAATTGTAAGCATTTTGCCATCCATACTTCAT   2698
S1001-like   TTGAGGAATGCTTGTTTTTTGTTCCATATCAGGGTGGTATTCATATCTATGGCTGCCTTTCATCTCCTTAATTGTAAGCATTTTGCCATCCATACTTCAT   2696
S52-like     TTGAGGAATGCTTGTTTTTTGTTCCATATCAGGGTGGTATTCATATCTATGGCTGCCTTTCATCTCCTTAATTGTAAGCATTTTGCCATCCATACTTCAT   2696
Hardwickii   TTGAGGAATGCTTGTTTTTTGTTCCATATCAGGGTGGTATTCATATCTATGGCTGCCTTTCATCTCCTTAATTGTAAGCATTTTGCCATCCATACTTCAT   2698

S05-like     TTATTTTCAAGCAATAGTCTTGTTTCATTCTTCCTTCAACTTTGCCTCTTTTGTATAATGCAGATAATTCTATTGGTGGGAACAAAGCTCCATGTTATTA   2798
S1001-like   TTATTTTCAAGCAATAGTCTTGTTTCATTCTTCATTCAACTTTGCCTCTTTTGTATAATGCAGATAATTCTATTGGTGGGAACAAAGCTCCATGTTATTA   2796
S52-like     TTATTTTCAAGCAATAGTCTTGTTTCATTCTTCATTCAACTTTGCCTCTTTTGTATAATGCAGATAATTCTATTGGTGGGAACAAAGCTCCATGTTATTA   2796
Hardwickii   TTATTTTCAAGCAATAGTCTTGTTTCATTCTTCATTCAACTTTGCCTCTTTTGTATAATGCAGATAATTCTATTGGTGGGAACAAAGCTCCATGTTATTA   2798

S05-like     TAACTCATATGGGATTGACAATTCAAGAAAGGGGTCATGTTGTGAAGGGTGTTCCCGTCGTTCAGCCTCGGGATGACCTGTTTTGGTTTGGACGTCCACA   2898
S1001-like   TAACTCATATGGGATTGACAATTCAAGAAAGGGGTCATGTTGTGAAGGGTGTTCCGGTCGTTCAGCCTCGGGATGACCTGTTTTGGTTTGGACGTCCACA   2896
S52-like     TAACTCATATGGGATTGACAATTCAAGAAAGGGGTCATGTTGTGAAGGGTGTTCCGGTCGTTCAGCCTCGGGATGACCTGTTTTGGTTTGGACGTCCACA   2896
Hardwickii   TAACTCATATGGGATTGACAATTCAAGAAAGGGGTCATGTTGTGAAGGGTGTTCCCGTCGTTCAGCCTCGGGATGACCTGTTTTGGTTTGGACGTCCACA   2898

S05-like     ACTTATTCTCTTCCTGATCCACTTTGTTCTCTTTATGGTATTATTTTTAACAACGTTATTTATGTTTCCACCAATAATTTATTTTCCTCCCTGAGAAAAT   2998
S1001-like   ACTTATTCTCTTCCTGATCCACTTTGTTCTCTTTATGGTATTATTTTTAACAACGTTATTTATGTTTCCACCAATAATTTATTTTCCTCCCTGAGAAAAT   2996
S52-like     ACTTATTCTCTTCCTGATCCACTTTGTTCTCTTTATGGTATTATTTTTAACAACGTTATTTATGTTTCCACCAATAATTTATTTTCCTCCCTGAGAAAAT   2996
Hardwickii   ACTTATTCTCTTCCTGATCCACTTTGTTCTCTTTATGGTATTATTTTTAACAACGTTATTTATGTTTCCACCAATAATTTATTTTCCTCCCTGAGAAAAT   2998

S05-like     GACATTCATCCTGTGCTTGCAGAATGCATTTCAGCTTGCCTTCTTTGCTTGGACCACAGTAAGCAATTCCTTTGAGACCATTTTTCAATCCAACTTTAGA   3098
S1001-like   GACATTCATCCTGTGCTTGCAGAATGCATTTCAGCTTGCCTTCTTTGCTTGGACCACTGTAAGCAATTCCTTTGAGACCATTTTTCAATCCAACTTTAGA   3096
S52-like     GACATTCATCCTGTGCTTGCAGAATGCATTTCAGCTTGCCTTCTTTGCTTGGACCACTGTAAGCAATTCCTTTGAGACCATTTTTCAATCCAACTTTAGA   3096
Hardwickii   GACATTCATCCTGTGCTTGCAGAATGCATTTCAGCTTGCCTTCTTTGCTTGGACCACAGTAAGCAATTCCTTTGAGACCATTTTTCAATCCAACTTTAGA   3098
```

```
S05-like      AATTATCTACACTGAGAACCATATCAAGCAAACTTCCCCCTAATAATGTGTTTAATTCTCTTTCAGTATGCATTTAAGTGGATGGGTTGTTTCCATCAGC   3198
S1001-like    AATTATCTACACTGAGAACCATATCAAGCAAACTTCCCCCTAATAATGTGTTTAATTCTCTTTCAGTATGCATTTAAGTGGATGGGTTGTTTCCATCAGC   3196
S52-like      AATTATCTACACTGAGAACCATATCAAGCAAACTTCCCCCTAATAATGTGTTTAATTCTCTTTCAGTATGCATTTAAGTGGATGGGTTGTTTCCATCAGC   3196
Hardwickii    AATTATCTACACTGAGAACCATATCAAGCAAACTTCCCCCTAATAATGTGTTTAATTCTCTTTCAGTATGCATTTAAGTGGATGGGTTGTTTCCATCAGC   3198

S05-like      GAGTTGAAGATATTGTCATCAGACTCTCAATGGGGTAAGTTCACAATATGAACTGTAAACAACCTGCGTTTCATCCTATCTATATGAACCACGAACTGAA   3298
S1001-like    GAGTTGAAGATATTGTCATCAGACTCTCAATGGGGTAAGTTCACAATATGAACTGTAAACAACCTGCGTTTCATCCTATCTATATGAACCACGAACTGAA   3296
S52-like      GAGTTGAAGATATTGTCATCAGACTCTCAATGGGGTAAGTTCACAATATGAACTGTAAACAACCTGCGTTTCATCCTATCTATATGAACCACGAACTGAA   3296
Hardwickii    GAGTTGAAGATATTGTCATCAGACTCTCAATGGGGTAAGTTCACAATATGAACTGTAAACAACCTGCGTTTCATCCTATCTATATGAACCACGAACTGAA   3298

S05-like      ACCGAAATCGACAACTTTTACAGGGTTATCATACAAGTTCTCTGCAGTTATGTCACACTCCCACTCTATGCTTTGGTTACTCAGGTAATCATCAAGCAAA   3398
S1001-like    ACCGAAATCGACAACTTTTACAGGGTTATCATACAAGTTCTCTGCAGTTATGTCACACTCCCACTCTATGCTTTGGTTACTCAGGTAATCATCAAGCAAA   3396
S52-like      ACCGAAATCGACAACTTTTACAGGGTTATCATACAAGTTCTCTGCAGTTATGTCACACTCCCACTCTATGCTTTGGTTACTCAGGTAATCATCAAGCAAA   3396
Hardwickii    ACCGAAATCGACAACTTTTACAGGGTTATCATACAAGTTCTCTGCAGTTATGTCACACTCCCACTCTATGCTTTGGTTACTCAGGTAATCATCAAGCAAA   3398

S05-like      AATTCCTTCTATTTTACTCTTCATCCTTCTTAAGATCATTGATCTGTTTTCTTGAGAAAAATTGAACTCAAAATAGTTCCAATAGAGAATAGCGACTCAT   3498
S1001-like    AATTCCTTCTATTTTACTCTTCATCCTTCTTAAGATCATTGATCTGTTTTCTTGAGAAAAATTGAACCCAAAATAGTTCCAATAGAGAATAGCGACTCAT   3496
S52-like      AATTCCTTCTATTTTACTCTTCATCCTTCTTAAGATCATTGATCTGTTTTCTTGAGAAAAATTGAACCCAAAATAGTTCCAATAGAGAATAGCGACTCAT   3496
Hardwickii    AATTCCTTCTATTTTACTCTTCATCCTTCTTAAGATCATTGATCTGTTTTCTTGAGAAAAATTGAACTCAAAATAGTTCCAATAGAGAATAGCGACTCAT   3498

S05-like      TACCTTGTGTGATTTTTTATATTCCAGATGGGCTCTAACATGAGACCAACCATTTTCAACGACCGAGTGGCGACGGCATTGAAGAACTGGCACCACTCAG   3598
S1001-like    TACCTTGTGTGATTTTTTATATTCCAGATGGGCTCTAACATGAGACCAACCATTTTCAACGACCGAGTGGCAACGGCATTGAAGAACTGGCACCACTCCG   3596
S52-like      TACCTTGTGTGATTTTTTATATTCCAGATGGGCTCTAACATGAGACCAACCATTTTCAACGACCGAGTGGCAACGGCATTGAAGAACTGGCACCACTCCG   3596
Hardwickii    TACCTTGTGTGATTTTTTATATTCCAGATGGGCTCTAACATGAGACCAACCATTTTCAACGACCGAGTGGCGACGGCATTGAAGAACTGGCACCACTCAG   3598

S05-like      CCAAGAAGAACATGAAGCAGCACCGCAACCCAGACAGTACCTCACCATTCTCAAGCAGGCCAGCTACTCCAACTCACGGCATGTCTCCTATTCACCTTCT   3698
S1001-like    CCAAGAAGAACATGAAGCAGCACCGCAACCCAGACAGTACCTCACCATTCTCAAGCAGGCCAGCTACTCCAACTCACGGCATGTCTCCTATTCACCTTCT   3696
S52-like      CCAAGAAGAACATGAAGCAGCACCGCAACCCAGACAGTACCTCACCATTCTCAAGCAGGCCAGCTACTCCAACTCACGGCATGTCTCCTATTCACCTTCT   3696
Hardwickii    CCAAGAAGAACATGAAGCAGCACCGCAACCCAGACAGTACCTCACCATTCTCAAGCAGGCCAGCTACTCCAACTCACGGCATGTCTCCTATTCACCTTCT   3698

S05-like      GCACAAACATCAGCATGGCAGCACATCTCCCAGGCTATCCGATGCCGAACCCGATCGTTGGGAAGAGTTGCCTCCTTCTTCACACCATAGTAGAGCCCCC   3798
S1001-like    GCACAAACATCAGCATGGCAGCACATCTCCCAGGCTATCCGATGCCGAACCCGATCGTTGGGAAGAGTTGCCTCCTTCTTCACACCATAGTAGAGCCCCC   3796
S52-like      GCACAAACATCAGCATGGCAGCACATCTCCCAGGCTATCCGATGCCGAACCCGATCGTTGGGAAGAGTTGCCTCCTTCTTCACACCATAGTAGAGCCCCC   3796
Hardwickii    GCACAAACATCAGCATGGCAGCACATCTCCCAGGCTATCCGATGCCGAACCCGATCGTTGGGAAGAGTTGCCTCCTTCTTCACACCATAGTAGAGCCCCC   3798

S05-like      CATCATGATAATCATCAAGATCAACAAGAACAATCTGAGACAATAATTAGAGAACAGGAGATGACAGTTCAAGGACCAAGTTCAAGTGAAACCGGTTCCA   3898
S1001-like    CATCATGATAATCATCAAGATCAACAAGAACAATCTGAGACAATAATTAGAGAACAGGAGATGACAGTTCAAGGACCAAGTTCAAGTGAAACCGGTTCCA   3896
S52-like      CATCATGATAATCATCAAGATCAACAAGAACAATCTGAGACAATAATTAGAGAACAGGAGATGACAGTTCAAGGACCAAGTTCAAGTGAAACCGGTTCCA   3896
Hardwickii    CATCATGATAATCATCAAGATCAACAAGAACAATCTGAGACAATAATTAGAGAACAGGAGATGACAGTTCAAGGACCAAGTTCAAGTGAAACCGGCTCCA   3898

S05-like      TAACACGTCCTGCTCGCCCTCATCAGGAAATCACTAGGACTCCATCAGACTTCTCATTTGCCAAATGA                                   3966
S1001-like    TAACACGTCCTGCTCGCCCTCATCAGGAAATCACTAGGACTCCATCAGACTTCTCATTTGCCAAATGA                                   3964
S52-like      TAACACGTCCTGCTCGCCCTCATCAGGAAATCACTAGGACTCCATCAGACTTCTCATTTGCCAAATGA                                   3964
Hardwickii    TAACACGTCCTGCTCGCCCTCATCAGGAAATCACTAGGACTCCATCAGACTTCTCATTTGCCAAATGA                                   3966
```

B

```
S05-like    MAECGTEQRTLEDTSTWAVAVVCFFLVVISIFIEHVIHLTGKWLEKRHKPALVEALEKVKAELMLLGFISLLLTIGQDAVTQICVSKELAATWLPCAARA    100
S52-like    MAECGTEQRTLEDTSTWAVAVVCFFLVVISIFIEHVIHLTGKWLEKRHKPALVEALEKVKAELMLLGFISLLLTIGQDAVTQICVSKELAATWLPCAARA    100

S05-like    KTGVKVAKNSRLRLLEFLDPDYGSRRILASKGDDACAKRGQLAFVSAYGIHQLHIFIFVLAVFHVLYCIITLAFGRTKMSKWKAWEDETKTIEYQYYNDP    200
S52-like    KTGVKVAKNSRLRLLEFLDPDYGSRRILASKGDDACAKRGQLAFVSAYGIHQLHIFIFVLAVFHVLYCIITLAFGRTKMSKWKAWEDETKTIEYQYYNDP    200

S05-like    ARFRFARDTTFGRRHLSFWSRTPISLWIVCFFRQFFGSVTKVDYMTLRHGFIVAHLAPGSEVKFDFHKYISRSLEDDFKVVVGISPAMWLFAVLFILTNT    300
S52-like    ARFRFARDTTFGRRHLSFWSRTPISLWIVCFFRQFFGSITKVDYMTLRHGFIVAHLAPGSEVKFDFHKYISRSLEDDFKVVVGISPAMWLFAVLFILTNT    300

S05-like    NGWYSYLWLPFISLIIIILLVGTKLHVIITHMGLTIQERGHVVKGVPVVQPRDDLFWFGRPQLILFLIHFVLFMNAFQLAFFAWTTYAFKWMGCFHQRVED    400
S52-like    NGWYSYLWLPFISLIIIILLVGTKLHVIITHMGLTIQERGHVVKGVPVVQPRDDLFWFGRPQLILFLIHFVLFMNAFQLAFFAWTTYAFKWMGCFHQRVED    400

S05-like    IVIRLSMGVIIQVLCSYVTLPLYALVTQMGSNMRPTIFNDRVATALKNWHHSAKKNMKQHRNPDSTSPFSSRPATPTHGMSPIHLLHKHQHGSTSPRLSD    500
S52-like    IVIRLSMGVIIQVLCSYVTLPLYALVTQMGSNMRPTIFNDRVATALKNWHHSAKKNMKQHRNPDSTSPFSSRPATPTHGMSPIHLLHKHQHGSTSPRLSD    500

S05-like    AEPDRWEELPPSSHHSRAPHHDNHQDQQEQSETIIREQEMTVQGPSSSETGSITRPARPHQEITRTPSDFSFAK                              574
S52-like    AEPDRWEELPPSSHHSRAPHHDNHQDQQEQSETIIREQEMTVQGPSSSETGSITRPARPHQEITRTPSDFSFAK                              574
```

Supplementary Figure 3. Alignment of the DNA and predicted amino acid sequences of the *CsMLO1* alleles from the susceptible cucumber inbred lines (Table S1). (A) Multiple sequence alignment of DNA sequences. *S05-like* represents the lines S05, 2003502, PI197088-S, True Lemon and Straight.Eight; *S1001-like* represents the lines S1001 and *gl*; *S52-like* denotes the lines S52 and 316; *Hardwickii* is the wild cucumber line Hardwickii. **(B)** Multiple sequence alignment of the predicted amino acid sequences. *S05-like* represents the lines S05, S1001, 2003502, PI197088-S, True Lemon, Straight.Eight, *gl* and Hardwickii; *S52-like* denotes the lines S52 and 316.

A

```
Haplotype A   ATTATTGTTGATTTTATGGAATCAATCTATATTTCATTCATTACTTAATAGAGATACAAGCCTATATATAACCATAGAGAAATACACTTAAGGAAATAAT   100
S94           ATTATTGTTGATTTTATGGAATCAATCTATATTTCATTCATTACTTAATAGAGATACAAGCCTATATATAACCATAGAGAAATACACTTAAGGAAATAAT   100
CGN20854      CCTTTTGTTGATTTTATGGAATCAATCTATATTTCATTAATTACTTAATAGAGATACAAGCCTATATATAACCATAGAGAAATACACTTAAGGAAATAAT   100

Haplotype A   ATCAAATAATATCTCCTAA............GAATAATATCTCCTAAGAATAATATCTCCTAAGAATAATATCTCCTAAGAATAATCTAATTATATTAAT   188
S94           ATCAAATAATATCTCCTAA............GAATAATATCTCCTAAGAATAATATCTCCTAAGAATAATATCTCCTAAGAATAATCTAATTATATTAAT   188
CGN20854      ATCAAATAATATCTCCAGAATAATATCTCCAGAATAATATCTCCTAAGAATAATATCTCCTAAGAATAATATCTCCTAAGAATAATCTAATTATATTAAT   200

Haplotype A   ACCCTCCCTCAAACTCAAGGTTGAAATCACAAACTTGAGTTTGCTAAGAACTAAGAAAAGAAACAATATATCTAGAATAGAATAAAAAACCCGAAGAACA   288
S94           ACCCTCCCTCAAACTCAAGGTTGAAATCACAAACTTGAGTTTGCTAAGAACTAAGAAAAGAAACAATATATCTAGAATAGAATAAAAAACCCGAAGAACA   288
CGN20854      ACCCTCCCTCAAACTCAAGGTTGAAATCACAAACTTGAGTTTGCTAAGAACTAAGAAAAGAAACAATATATCTAGAATAGAATAAAAAACCCGAAGAACA   300

Haplotype A   AACACACAAAGAACTTCTAGGCTAAAACAAACCCACGAAGAGCGAAACGAAGAACTTCTGGGATAGAAACATAGATCCTCGTATAGAGATCTATAACAGA   388
S94           AACACACAAAGAACTTCTAGGCTAAAACAAACCCACGAAGAGCGAAACGAAGAACTTCTGGGATAGAAACATAGATCCTCGTATAGAGATCTATAACAGA   388
CGN20854      AACACACAAAGAACTTCTAGGCTAAAACAAACCCACGA.GAGCGAAACGAAGAACTTCTGGGATAGAAACATAGATCCTCGTATAGAGATCTATAACAGA   399

Haplotype A   ACCTAGGAAGAACGAAACAAGAACTCCGGGGGCAAAATGAAAGAGCAAAACTGAAGCAAAGTAGAATCAAATCAGGATGAAAACGTGGTCTGAATTGGGC   488
S94           ACCTAGGAAGAACGAAACAAGAACTCCGGGGGCAAAATGAAAGAGCAAAACTGAAGCAAAGTAGAATCAAATCAGGATGAAAACGTGGTCTGAATTGGGC   488
CGN20854      ACCTAGGAAGAACGAAACAAGAACTCCGGGGGCAAAATGAAAGAGCAAAACTGAAGCAAAGTAGAA.CAAATCAGGATGAAAACGTGGTCTGAATTGGGC   498

Haplotype A   AGGAGCGTTTCGTCATCAGAATGGAACTGAACGAACTAAACTAAAGCAAGTCAGAATCAAACTGAACAGGAAATCTGGATGAACAGAACGAAAACTGAAG   588
S94           AGGAGCGTTTCGTCATCAGAATGGAACTGAACGAACTAAACTAAAGCAAGTCAGAATCAAACTGAACAGGAAATCTGGATGAACAGAACGAAAACTGAAG   588
CGN20854      AGGAGCGTTTCGTCATCAGAATGGAACTGAACGAACTAAACTAAAGCAAGTCAGAATCAAACTGAACAGGAAATCTGGGTGAACAGAACGAAAACTGAAG   598

Haplotype A   AGAACTGAGAGGTTGAGTAGATAGAGAACTTTGCGGCGTTCTTCGCAAAAAGAGAAGATGCGTTTCATGACGGCGAGCTTCACAAATGGAGAGCGGACAA   688
S94           AGAACTGAGAGGTTGAGTAGATAGAGAACTTTGCGGCGTTCTTCGCAAAAAGAGAAGATGCGTTTCATGACGGCGAGCTTCACAAATGGAGAGCGGACAA   688
CGN20854      AGAGCTGAGAGGTTGAGTAGATAGAGAGCTTTGTGGCGTTCTTCGCAAAAAGAGAAGATGCGTTTCATGACGGCGAGCTTCACAAATGGAGAGCGGACAA   698

Haplotype A   AGCCGAACCTGTGCGTCGTCGGCTGTGAGTGGAGCGGAGCGGTGGATCATGGATCTGAACAAAGATTCACCCTCTTCAGGATCGGGAGTCTTCAATCTAA   788
S94           AGCCGAACCTGTGCGTCGTCGGCTGTGAGTGGAGCGGAGCGGTGGATCATGGATCTGAACAAAGATTCACCCTCTTCAGGATCGGGAGTCTTCAATCTAA   788
CGN20854      AGCCGAACCTGTGCGTCGTCGGCTGTGAGTGGAGCGGAGCGGTGGATCATGGATCTGAACAAAGATTCACCCTCTTCAGGATCGGGAGTCTTCAATCTAA   798

Haplotype A   AGAGATTGTCTGGTACGAGTAAAGGGCGGCGGCGGCAGGTCGGTCTGATCTTGAACAGTATGATTCGAAGGCGAAAGATGAAATAAAACACGGATCTGGA   888
S94           AGAGATTGTCTGGTACGAGTAAAGGGCGGCGGCGGCAGGTCGGTCTGATCTTGAACAGTATGATTCGAAGGCGAAAGATGAAATAAAACACGGATCTGGA   888
CGN20854      AGAGATTGTCTGGTACGAGTAAAGGGCGGCGGCGGCAGGTCGGTCTGATCTTGAACAGTATGATTCGAAGGCGAAAGATGAAATAAAACACGGATCTGGA   898

Haplotype A   AGTCGAAGACACGAACAGAGATGCACGAACAAATCGGTGCTTGCAATGTGCGCACAAGTAGATCTAAGTGAGGACGAATGGAGAGCGGATATGGGTCGAG   988
S94           AGTCGAAGACACGAACAGAGATGCACGAACAAATCGGTGCTTGCAATGTGCGCACAAGTAGATCTAAGTGAGGACGAATGGAGAGCGGATATGGGTCGAG   988
CGN20854      AGTCGAAGACACGAACAGAGATGCACGAACAAATCGGTGCTTGCAATGTGCGCACAAGTAGATCTAAGTGAGGACGAATGGAGAGCGGATATGGGTCGAG   998

Haplotype A   AGTGCGACGAACTGGGTCGAGAGTGCGACGAACTGTGTGGATGGAAGGAGCAGTGATGGCGGAAGGAGCAGTGAT..................GGCAGAA   1070
S94           AGTGCGACGAACTGGGTCGAGAGTGCGACGAACTGTGTGGATGGAAGGAGCAGTGATGGCGGAAGGAGCAGTGATGGCGGAAGGAGCAGTGATGGCAGAA   1088
CGN20854      AGT.....................GCGACGAACTGTGTGGATGGAAGGAGCAGTGATGGCGGAAGGAGCAGTGAT..................GGCAGAA   1059

Haplotype A   GGAGTAGTGGCGGCGGCAGCAGAATCAGAGACGAGGATTTTGATCTAGGAGGCAGAGCGAATTACTTGGAGACATGATGTGTATTGATTACTGATGGGTA   1170
S94           GGAGTAGTGGCGGCGGCAGCAGAATCAGAGACGAGGATTTTGATCTAGGAGGCAGAGCGAATTACTTGGAGACATGATGTGTATTGATTACTGATGGGTA   1188
CGN20854      GGAGTAGTGGCGGCGGCGGCAGAATCAGAGACGAGGATTTTGATCTAGGAGGCAGAGCGAATTACTTGGAGACATGATGTGTATTGATTACTGATGGGTA   1159

Haplotype A   TGGCTCTGCCTCCGTCGACGAATCAACGGAGGCAGACGAGCGGGGATGTGACGGCTAGAATACAAGGAGACT.AAAACCCTATAGCTCTGATACCATGTT   1269
S94           TGGCTCTGCCTCCGTCGACGAATCAACGGAGGCAGACGAGCGGGGATGTGACGGCTAGAATACAAGGAGACT.AAAACCCTATAGCTCTGATACCATGTT   1287
CGN20854      TGGCTCTGCCTCCGTCGACGAATCAACGGAGGCAGACGTGCAGGGATGTGACGGCTAGAATACAAGGAGACTTAAAATCCTATAGCTCTGATACCATGTT   1259

Haplotype A   GATTTTATGGAATCAATCTATATTTCATTCATTACTTAATAGAGATACAAGCCTATATATAACCATAGAGAAATACACTTAAGGAAATAATATCAAATAA   1369
S94           GATTTTATGGAATCAATCTATATTTCATTCATTACTTAATAGAGATACAAGCCTATATATAACCATAGAGAAATACACTTAAGGAAATAATATCAAATAA   1387
CGN20854      GATTTTATGGAATCAATCTATATTTCATTAATTACTTAATAGAGATACAAGCCTATATATAACCATAGAGAAATACACTTAAGGAAATAATATCAAATAA   1359

Haplotype A   TATCTCCTAA............GAATAATATCTCCTAAGAATAATATCTCCTAAGAATAATATCTCCTAAGAATAATCTAATTATATTAATAATTAT   1454
S94           TATCTCCTAA............GAATAATATCTCCTAAGAATAATATCTCCTAAGAATAATATCTCCTAAGAATAATCTAATTATATTAATAATTAT   1472
CGN20854      TATCTCCAGAATAATATCTCCAGAATAATATCTCCTAAGAATAATATCTCCTAAGAATAATATCTCCTAAGAATAATCTAATTATATTAATACCTTT   1456
```

B

```
First 189bp in Haplotype A    ATTATTGTTGATTTTATGGAATCAATCTATATTTCATTCATTACTTAATAGAGATACAAGCCTATATATAACCATAGAGA    80
Last 189bp in Haplotype A     .....TGTTGATTTTATGGAATCAATCTATATTTCATTCATTACTTAATAGAGATACAAGCCTATATATAACCATAGAGA    75
First 201bp in CGN20854       CCTTTTGTTGATTTTATGGAATCAATCTATATTTCATTAATTACTTAATAGAGATACAAGCCTATATATAACCATAGAGA    80
Last 201bp in CGN20854        .....TGTTGATTTTATGGAATCAATCTATATTTCATTAATTACTTAATAGAGATACAAGCCTATATATAACCATAGAGA    75

First 189bp in Haplotype A    AATACACTTAAGGAAATAATATCAAATAATATCTCCTA............AGAATAATATCTCCTAAGAATAATATCTCC   148
Last 189bp in Haplotype A     AATACACTTAAGGAAATAATATCAAATAATATCTCCTA............AGAATAATATCTCCTAAGAATAATATCTCC   143
First 201bp in CGN20854       AATACACTTAAGGAAATAATATCAAATAATATCTCCAGAATAATATCTCCAGAATAATATCTCCTAAGAATAATATCTCC   160
Last 201bp in CGN20854        AATACACTTAAGGAAATAATATCAAATAATATCTCCAGAATAATATCTCCAGAATAATATCTCCTAAGAATAATATCTCC   155

First 189bp in Haplotype A    TAAGAATAATATCTCCTAAGAATAATCTAATTATATTAATA.....                                    189
Last 189bp in Haplotype A     TAAGAATAATATCTCCTAAGAATAATCTAATTATATTAATAATTAT                                    189
First 201bp in CGN20854       TAAGAATAATATCTCCTAAGAATAATCTAATTATATTAATA.....                                    201
Last 201bp in CGN20854        TAAGAATAATATCTCCTAAGAATAATCTAATTATATTAATACCTTT                                    201
```

Supplementary Figure 4. Comparison of the insertion sequences in *CsMLO1* between haplotype A, CGN20854 and S94. (A) Multiple sequence alignment of the entire insertion sequences plus the target site duplication (TSD). **(B)** Multiple sequence alignment of the first and last long terminal repeat (LTR) parts of the insertion sequences plus the target site duplication (TSD) in haplotype A and CGN20854.

A

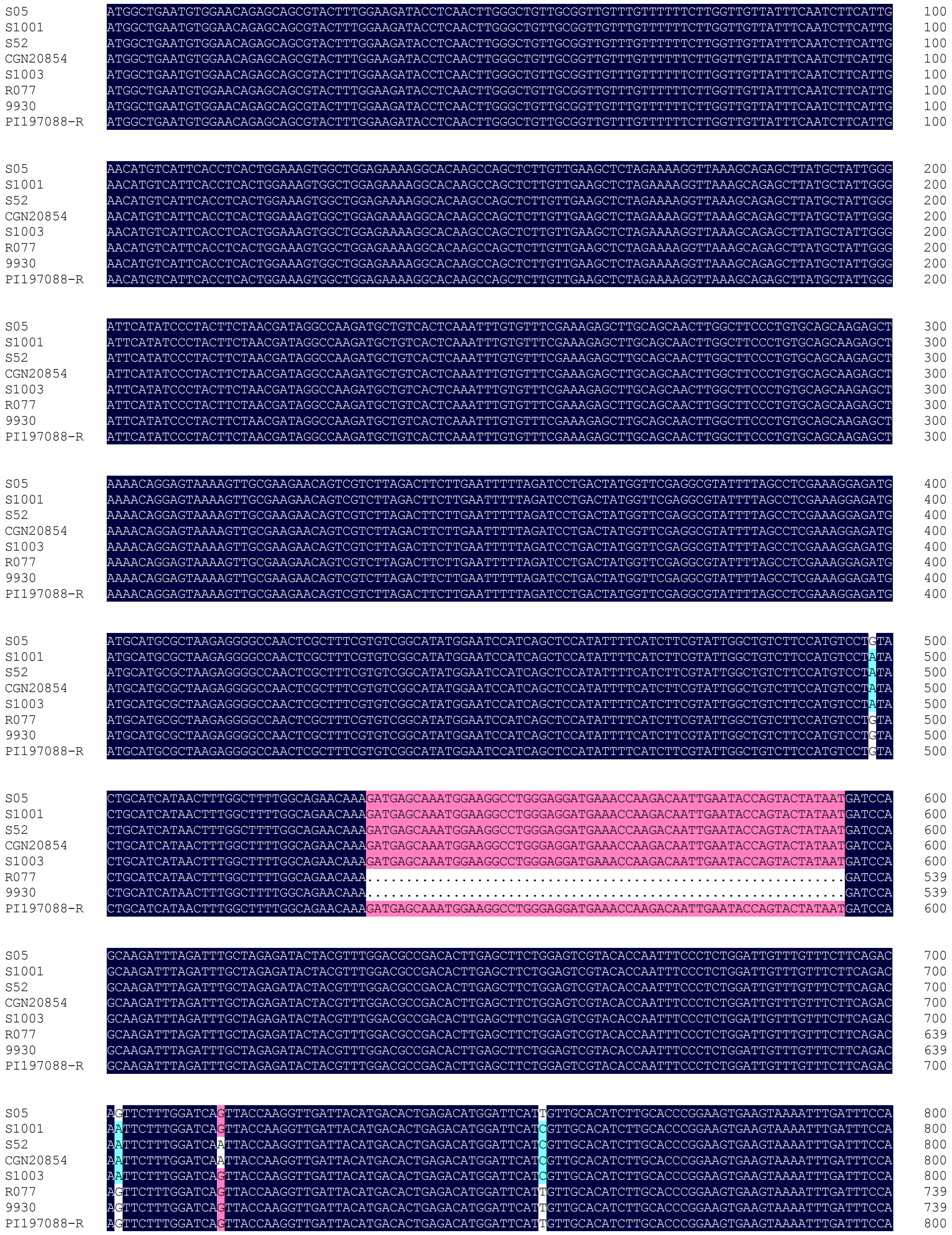

```
S05         CAAATACATTAGCAGATCTCTGGAAGACGACTTTAAAGTTGTTGTGGGGATTAGTCCCGCAATGTGGCTATTTGCTGTTCTCTTCATCCTAACCAATACA   900
S1001       CAAATACATTAGCAGATCTCTGGAAGACGACTTTAAAGTTGTTGTGGGGATTAGTCCCGCAATGTGGCTATTTGCTGTTCTCTTCATCCTAACCAATACA   900
S52         CAAATACATTAGCAGATCTCTGGAAGACGACTTTAAAGTTGTTGTGGGGATTAGTCCCGCAATGTGGCTATTTGCTGTTCTCTTCATCCTAACCAATACA   900
CGN20854    CAAATACATTAGCAGATCTCTGGAAGACGACTTTAAAGTTGTTGTGGGGATTAGTCCCGCAATGTGGCTATTTGCTGTTCTCTTCATCCTAACCAATACA   900
S1003       CAAATACATTAGCAGATCTCTGGAAGACGACTTTAAAGTTGTTGTGGGGATTAGTCCCGCAATGTGGCTATTTGCTGTTCTCTTCATCCTAACCAATACA   900
R077        CAAATACATTAGCAGATCTCTGGAAGACGACTTTAAAGTTGTTGTGGGGATTAGTCCCGCAATGTGGCTATTTGCTGTTCTCTTCATCCTAACCAATACA   839
9930        CAAATACATTAGCAGATCTCTGGAAGACGACTTTAAAGTTGTTGTGGGGATTAGTCCCGCAATGTGGCTATTTGCTGTTCTCTTCATCCTAACCAATACA   839
PI197088-R  CAAATACATTAGCAGATCTCTGGAAGACGACTTTAAAGTTGTTGTGGGGATTAGTCCCGCAATGTGGCTATTTGCTGTTCTCTTCATCCTAACCAATACA   900

S05         AATGGGTGGTATTCATATCTATGGCTGCCTTTCATCTCCTTAATTATAATTCTATTGGTGGGAACAAAGCTCCATGTTATTATATAACTCATATGGATTGA   1000
S1001       AATGGGTGGTATTCATATCTATGGCTGCCTTTCATCTCCTTAATTATAATTCTATTGGTGGGAACAAAGCTCCATGTTATTATATAACTCATATGGATTGA   1000
S52         AATGGGTGGTATTCATATCTATGGCTGCCTTTCATCTCCTTAATTATAATTCTATTGGTGGGAACAAAGCTCCATGTTATTATATAACTCATATGGATTGA   1000
CGN20854    AATGGGTGGTATTCATATCTATGGCTGCCTTTCATCTCCTTAATTATAATTCTATTGGTGGGAACAAAGCTCCATGTTATTATATAACTCATATGGATTGA   1000
S1003       AATGGGTGGTATTCATATCTATGGCTGCCTTTCATCTCCTTAATT.......................................................   945
R077        AATGGGTGGTATTCATATCTATGGCTGCCTTTCATCTCCTTAATTATAATTCTATTGGTGGGAACAAAGCTCCATGTTATTATATAACTCATATGGATTGA   939
9930        AATGGGTGGTATTCATATCTATGGCTGCCTTTCATCTCCTTAATTATAATTCTATTGGTGGGAACAAAGCTCCATGTTATTATATAACTCATATGGATTGA   939
PI197088-R  AATGGGTGGTATTCATATCTATGGCTGCCTTTCATCTCCTTAATTATAATTCTATTGGTGGGAACAAAGCTCCATGTTATTATATAACTCATATGGATTGA   1000

S05         CAATTCAAGAAAGGGGTCATGTTGTGAAGGGTGTTCCCGTCGTTCAGCCTCGGGATGACCTGTTTTGGTTTGGACGTCCACAACTTATTCTCTTCCTGAT   1100
S1001       CAATTCAAGAAAGGGGTCATGTTGTGAAGGGTGTTCCGGTCGTTCAGCCTCGGGATGACCTGTTTTGGTTTGGACGTCCACAACTTATTCTCTTCCTGAT   1100
S52         CAATTCAAGAAAGGGGTCATGTTGTGAAGGGTGTTCCGGTCGTTCAGCCTCGGGATGACCTGTTTTGGTTTGGACGTCCACAACTTATTCTCTTCCTGAT   1100
CGN20854    CAATTCAAGAAAGGGGTCATGTTGTGAAGGGTGTTCCGGTCGTTCAGCCTCGGGATGACCTGTTTTGGTTTGGACGTCCACAACTTATTCTCTTCCTGAT   1100
S1003       ....................................................................................................   945
R077        CAATTCAAGAAAGGGGTCATGTTGTGAAGGGTGTTCCCGTCGTTCAGCCTCGGGATGACCTGTTTTGGTTTGGACGTCCACAACTTATTCTCTTCCTGAT   1039
9930        CAATTCAAGAAAGGGGTCATGTTGTGAAGGGTGTTCCCGTCGTTCAGCCTCGGGATGACCTGTTTTGGTTTGGACGTCCACAACTTATTCTCTTCCTGAT   1039
PI197088-R  CAATTCAAGAAAGGGGTCATGTTGTGAAGGGTGTTCCCGTCGTTCAGCCTCGGGATGACCTGTTTTGGTTTGGACGTCCACAACTTATTCTCTTCCTGAT   1100

S05         CCACTTTGTTCTCTTATGAATGCATTTCAGCTTGCCTTCTTTGCTTGGACCACATATGCATTTAAGTGGATGGGTTGTTTCCATCAGCGAGTTGAAGAT   1200
S1001       CCACTTTGTTCTCTTATGAATGCATTTCAGCTTGCCTTCTTTGCTTGGACCACTTATGCATTTAAGTGGATGGGTTGTTTCCATCAGCGAGTTGAAGAT   1200
S52         CCACTTTGTTCTCTTATGAATGCATTTCAGCTTGCCTTCTTTGCTTGGACCACTTATGCATTTAAGTGGATGGGTTGTTTCCATCAGCGAGTTGAAGAT   1200
CGN20854    CCACTTTGTTCTCTTATGAATGCATTTCAGCTTGCCTTCTTTGCTTGGACCACTTATGCATTTAAGTGGATGGGTTGTTTCCATCAGCGAGTTGAAGAT   1200
S1003       ..................AATGCATTTCAGCTTGCCTTCTTTGCTTGGACCACTTATGCATTTAAGTGGATGGGTTGTTTCCATCAGCGAGTTGAAGAT   1026
R077        CCACTTTGTTCTCTTATGAATGCATTTCAGCTTGCCTTCTTTGCTTGGACCACATATGCATTTAAGTGGATGGGTTGTTTCCATCAGCGAGTTGAAGAT   1139
9930        CCACTTTGTTCTCTTATGAATGCATTTCAGCTTGCCTTCTTTGCTTGGACCACATATGCATTTAAGTGGATGGGTTGTTTCCATCAGCGAGTTGAAGAT   1139
PI197088-R  CCACTTTGTTCTCTTATGAATGCATTTCAGCTTGCCTTCTTTGCTTGGACCACATATGCATTTAAGTGGATGGGTTGTTTCCATCAGCGAGTTGAAGAT   1200

S05         ATTGTCATCAGACTCTCAATGGGGGTTATCATACAAGTTCTCTGCAGTTATGTCACACTCCCACTCTATGCTTTGGTTACTCAGATGGGCTCTAACATGA   1300
S1001       ATTGTCATCAGACTCTCAATGGGGGTTATCATACAAGTTCTCTGCAGTTATGTCACACTCCCACTCTATGCTTTGGTTACTCAGATGGGCTCTAACATGA   1300
S52         ATTGTCATCAGACTCTCAATGGGGGTTATCATACAAGTTCTCTGCAGTTATGTCACACTCCCACTCTATGCTTTGGTTACTCAGATGGGCTCTAACATGA   1300
CGN20854    ATTGTCATCAGACTCTCAATGGGGGTTATCATACAAGTTCTCTGCAGTTATGTCACACTCCCACTCTATGCTTTGGTTACTCAGATGGGCTCTAACATGA   1300
S1003       ATTGTCATCAGACTCTCAATGGGGGTTATCATACAAGTTCTCTGCAGTTATGTCACACTCCCACTCTATGCTTTGGTTACTCAGATGGGCTCTAACATGA   1126
R077        ATTGTCATCAGACTCTCAATGGGGGTTATCATACAAGTTCTCTGCAGTTATGTCACACTCCCACTCTATGCTTTGGTTACTCAGATGGGCTCTAACATGA   1239
9930        ATTGTCATCAGACTCTCAATGGGGGTTATCATACAAGTTCTCTGCAGTTATGTCACACTCCCACTCTATGCTTTGGTTACTCAGATGGGCTCTAACATGA   1239
PI197088-R  ATTGTCATCAGACTCTCAATGGGGGTTATCATACAAGTTCTCTGCAGTTATGTCACACTCCCACTCTATGCTTTGGTTACTCAGATGGGCTCTAACATGA   1300

S05         GACCAACCATTTTCAACGACCGAGTGGCGACGGCATTGAAGAACTGGCACCACTCAGCCAAGAAGAACATGAAGCAGCACCGCAACCCAGACAGTACCTC   1400
S1001       GACCAACCATTTTCAACGACCGAGTGGCAACGGCATTGAAGAACTGGCACCACTCCGCCAAGAAGAACATGAAGCAGCACCGCAACCCAGACAGTACCTC   1400
S52         GACCAACCATTTTCAACGACCGAGTGGCAACGGCATTGAAGAACTGGCACCACTCCGCCAAGAAGAACATGAAGCAGCACCGCAACCCAGACAGTACCTC   1400
CGN20854    GACCAACCATTTTCAACGACCGAGTGGCAACGGCATTGAAGAACTGGCACCACTCCGCCAAGAAGAACATGAAGCAGCACCGCAACCCAGACAGTACCTC   1400
S1003       GACCAACCATTTTCAACGACCGAGTGGCAACGGCATTGAAGAACTGGCACCACTCCGCCAAGAAGAACATGAAGCAGCACCGCAACCCAGACAGTACCTC   1226
R077        GACCAACCATTTTCAACGACCGAGTGGCGACGGCATTGAAGAACTGGCACCACTCAGCCAAGAAGAACATGAAGCAGCACCGCAACCCAGACAGTACCTC   1339
9930        GACCAACCATTTTCAACGACCGAGTGGCGACGGCATTGAAGAACTGGCACCACTCAGCCAAGAAGAACATGAAGCAGCACCGCAACCCAGACAGTACCTC   1339
PI197088-R  GACCAACCATTTTCAACGACCGAGTGGCGACGGCATTGAAGAACTGGCACCACTCAGCCAAGAAGAACATGAAGCAGCACCGCAACCCAGACAGTACCTC   1400

S05         ACCATTCTCAAGCAGGCCAGCTACTCCAACTCACGGCATGTCTCCTATTCACCTTCTGCAC.AAACATCAGCATGGCAGCACATCTCCCAGGCTATCCGA   1499
S1001       ACCATTCTCAAGCAGGCCAGCTACTCCAACTCACGGCATGTCTCCTATTCACCTTCTGCAC.AAACATCAGCATGGCAGCACATCTCCCAGGCTATCCGA   1499
S52         ACCATTCTCAAGCAGGCCAGCTACTCCAACTCACGGCATGTCTCCTATTCACCTTCTGCAC.AAACATCAGCATGGCAGCACATCTCCCAGGCTATCCGA   1499
CGN20854    ACCATTCTCAAGCAGGCCAGCTACTCCAACTCACGGCATGTCTCCTATTCACCTTCTGCAC.AAACATCAGCATGGCAGCACATCTCCCAGGCTATCCGA   1499
S1003       ACCATTCTCAAGCAGGCCAGCTACTCCAACTCACGGCATGTCTCCTATTCACCTTCTGCAC.AAACATCAGCATGGCAGCACATCTCCCAGGCTATCCGA   1325
R077        ACCATTCTCAAGCAGGCCAGCTACTCCAACTCACGGCATGTCTCCTATTCACCTTCTGCAC.AAACATCAGCATGGCAGCACATCTCCCAGGCTATCCGA   1438
9930        ACCATTCTCAAGCAGGCCAGCTACTCCAACTCACGGCATGTCTCCTATTCACCTTCTGCAC.AAACATCAGCATGGCAGCACATCTCCCAGGCTATCCGA   1438
PI197088-R  ACCATTCTCAAGCAGGCCAGCTACTCCAACTCACGGCATGTCTCCTATTCACCTTCTGCACAAAACATCAGCATGGCAGCACATCTCCCAGGCTATCCGA   1500

S05         TGCCGAACCCGATCGTTGGGAAGAGTTGCCTCCTTCTTCACACCATAGTAGAGCCCCCCATCATGATAATCATCAAGATCAACAAGAACAATCTGAGACA   1599
S1001       TGCCGAACCCGATCGTTGGGAAGAGTTGCCTCCTTCTTCACACCATAGTAGAGCCCCCCATCATGATAATCATCAAGATCAACAAGAACAATCTGAGACA   1599
S52         TGCCGAACCCGATCGTTGGGAAGAGTTGCCTCCTTCTTCACACCATAGTAGAGCCCCCCATCATGATAATCATCAAGATCAACAAGAACAATCTGAGACA   1599
CGN20854    TGCCGAACCCGATCGTTGGGAAGAGTTGCCTCCTTCTTCACACCATAGTAGAGCCCCCCATCATGATAATCATCAAGATCAACAAGAACAATCTGAGACA   1599
S1003       TGCCGAACCCGATCGTTGGGAAGAGTTGCCTCCTTCTTCACACCATAGTAGAGCCCCCCATCATGATAATCATCAAGATCAACAAGAACAATCTGAGACA   1425
R077        TGCCGAACCCGATCGTTGGGAAGAGTTGCCTCCTTCTTCACACCATAGTAGAGCCCCCCATCATGATAATCATCAAGATCAACAAGAACAATCTGAGACA   1538
9930        TGCCGAACCCGATCGTTGGGAAGAGTTGCCTCCTTCTTCACACCATAGTAGAGCCCCCCATCATGATAATCATCAAGATCAACAAGAACAATCTGAGACA   1538
PI197088-R  TGCCGAACCCGATCGTTGGGAAGAGTTGCCTCCTTCTTCACACCATAGTAGAGCCCCCCATCATGATAATCATCAAGATCAACAAGAACAATCTGAGACA   1600

S05         ATAATTAGAGAACAGGAGATGACAGTTCAAGGACCAAGTTCAAGTGAAACCGGTTCATAACACGTCCTGCTCGCCCTCATCAGGAAATCACTAGGACTC   1699
S1001       ATAATTAGAGAACAGGAGATGACAGTTCAAGGACCAAGTTCAAGTGAAACCGGTTCATAACACGTCCTGCTCGCCCTCATCAGGAAATCACTAGGACTC   1699
S52         ATAATTAGAGAACAGGAGATGACAGTTCAAGGACCAAGTTCAAGTGAAACCGGTTCATAACACGTCCTGCTCGCCCTCATCAGGAAATCACTAGGACTC   1699
CGN20854    ATAATTAGAGAACAGGAGATGACAGTTCAAGGACCAAGTTCAAGTGAAACCGGTTCATAACACGTCCTGCTCGCCCTCATCAGGAAATCACTAGGACTC   1699
S1003       ATAATTAGAGAACAGGAGATGACAGTTCAAGGACCAAGTTCAAGTGAAACCGGTTCATAACACGTCCTGCTCGCCCTCATCAGGAAATCACTAGGACTC   1525
R077        ATAATTAGAGAACAGGAGATGACAGTTCAAGGACCAAGTTCAAGTGAAACCGGTTCATAACACGTCCTGCTCGCCCTCATCAGGAAATCACTAGGACTC   1638
9930        ATAATTAGAGAACAGGAGATGACAGTTCAAGGACCAAGTTCAAGTGAAACCGGTTCATAACACGTCCTGCTCGCCCTCATCAGGAAATCACTAGGACTC   1638
PI197088-R  ATAATTAGAGAACAGGAGATGACAGTTCAAGGACCAAGTTCAAGTGAAACCGGTTCATAACACGTCCTGCTCGCCCTCATCAGGAAATCACTAGGACTC   1700

S05         CATCAGACTTCTCATTTGCCAAATGA   1725
S1001       CATCAGACTTCTCATTTGCCAAATGA   1725
S52         CATCAGACTTCTCATTTGCCAAATGA   1725
CGN20854    CATCAGACTTCTCATTTGCCAAATGA   1725
S1003       CATCAGACTTCTCATTTGCCAAATGA   1551
R077        CATCAGACTTCTCATTTGCCAAATGA   1664
9930        CATCAGACTTCTCATTTGCCAAATGA   1664
PI197088-R  CATCAGACTTCTCATTTGCCAAATGA   1726
```

B

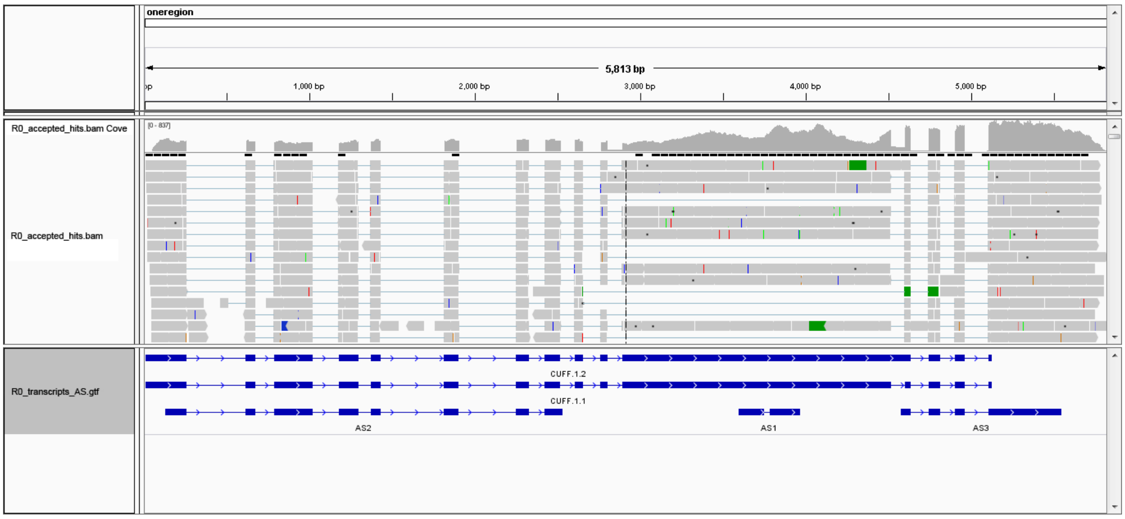

Supplementary Figure 5. The transcripts of the *CsMLO1* alleles. (A) Multiple sequence alignment of the *CsMLO1* allele transcripts in representative susceptible and resistant lines. **(B)** The *CsMLO1* allele transcripts in S1003 were obtained by assembling clean tags from transcriptome sequencing of S1003.

A

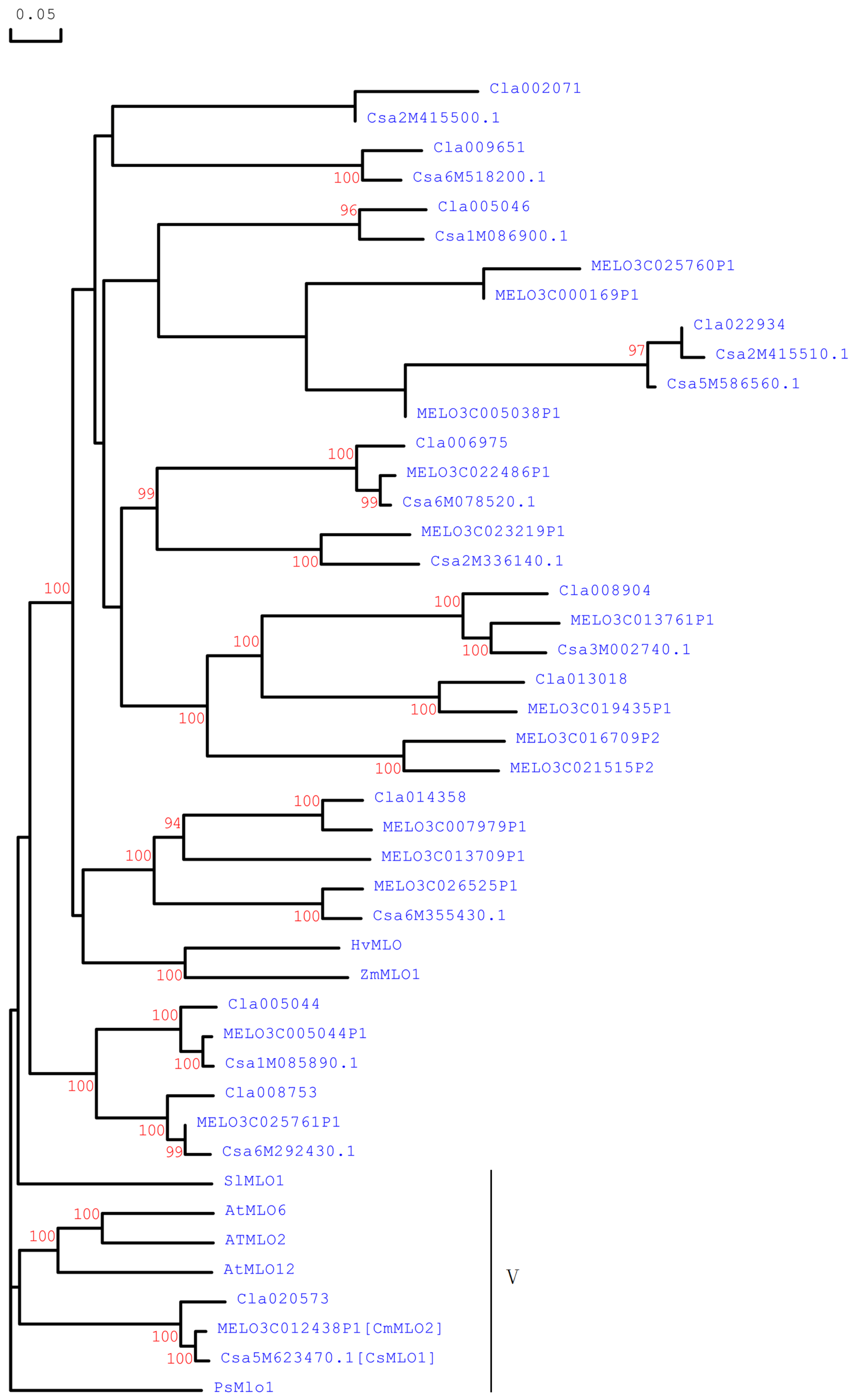
0.05
Cla002071
Csa2M415500.1
Cla009651
Csa6M518200.1
Cla005046
Csa1M086900.1
MELO3C025760P1
MELO3C000169P1
Cla022934
Csa2M415510.1
Csa5M586560.1
MELO3C005038P1
Cla006975
MELO3C022486P1
Csa6M078520.1
MELO3C023219P1
Csa2M336140.1
Cla008904
MELO3C013761P1
Csa3M002740.1
Cla013018
MELO3C019435P1
MELO3C016709P2
MELO3C021515P2
Cla014358
MELO3C007979P1
MELO3C013709P1
MELO3C026525P1
Csa6M355430.1
HvMLO
ZmMLO1
Cla005044
MELO3C005044P1
Csa1M085890.1
Cla008753
MELO3C025761P1
Csa6M292430.1
SlMLO1
AtMLO6
ATMLO2
AtMLO12
Cla020573
MELO3C012438P1[CmMLO2]
Csa5M623470.1[CsMLO1]
PsMlo1
V

B

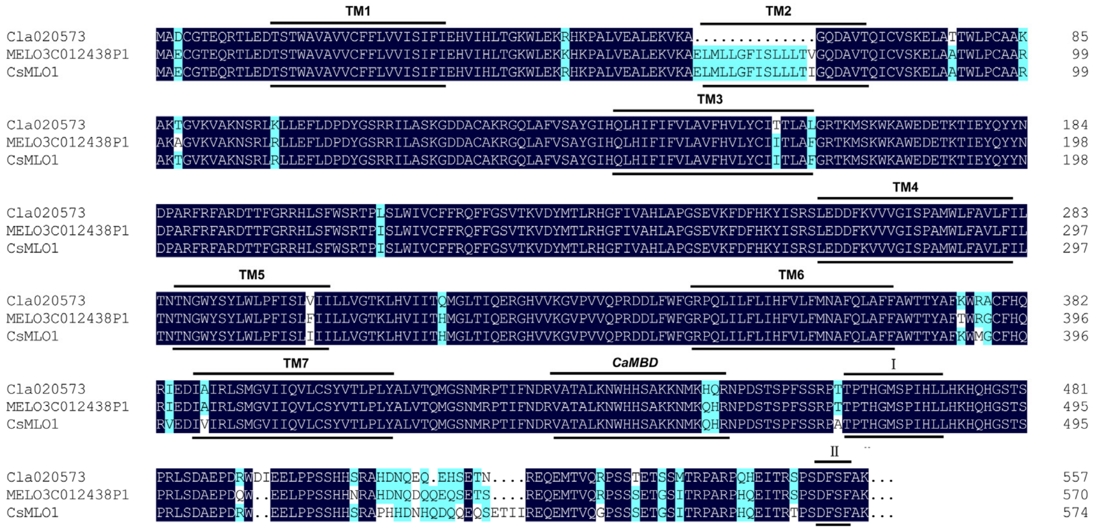

Supplementary Figure 6. Phylogenetic analysis of the MLO proteins in cucumber, melon and watermelon and multiple sequence alignment of the three MLOs clustered in clade V. (A) Phylogenetic tree analysis of the MLO proteins in cucumber, melon and watermelon with MLO proteins from other species, including HvMLO (barley), AtMLO2, AtMLO6, AtMLO12 (*Arabidopsis*), ZmMLO1 (maize), SlMLO1 (tomato) and PsMLO1 (pea). “Csa”, “MELO” and “Cla” denote the MLO family proteins from cucumber, melon and watermelon, respectively. Clade V was designated based on Devoto et al. (2003). Numbers at the nodes indicate bootstrap support based on 1000 replicates. **(B)** Multiple sequence alignment of the three MLOs (CsMLO1, MELO3C012438P1 and Cla020573) clustered in clade V. The positions of the transmembrane regions (TM1–TM7) inferred from the experimentally determined topology of barley HvMLO (Devoto et al., 1999), the approximate position of the CaMBD (Kim et al., 2002a, b) and the other two conserved domains (I and II) (Panstruga, 2005b) are indicated by lines above and below the aligned sequences.

A

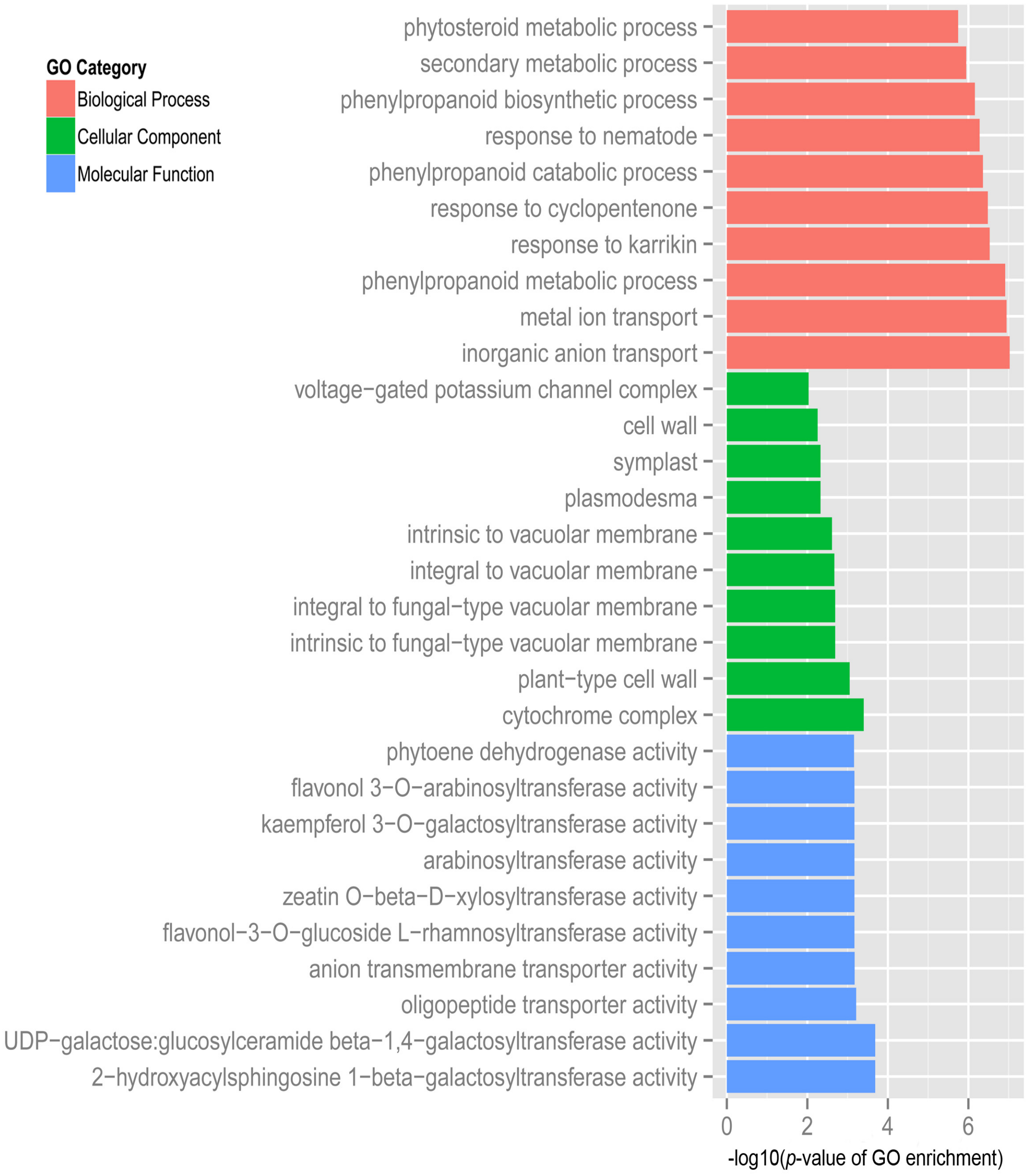
GO Category
Biological Process
Cellular Component
Molecular Function
phytosteroid metabolic process
secondary metabolic process
phenylpropanoid biosynthetic process
response to nematode
phenylpropanoid catabolic process
response to cyclopentenone
response to karrikin
phenylpropanoid metabolic process
metal ion transport
inorganic anion transport
voltage−gated potassium channel complex
cell wall
symplast
plasmodesma
intrinsic to vacuolar membrane
integral to vacuolar membrane
integral to fungal−type vacuolar membrane
intrinsic to fungal−type vacuolar membrane
plant−type cell wall
cytochrome complex
phytoene dehydrogenase activity
flavonol 3−O−arabinosyltransferase activity
kaempferol 3−O−galactosyltransferase activity
arabinosyltransferase activity
zeatin O−beta−D−xylosyltransferase activity
flavonol−3−O−glucoside L−rhamnosyltransferase activity
anion transmembrane transporter activity
oligopeptide transporter activity
UDP−galactose:glucosylceramide beta−1,4−galactosyltransferase activity
2−hydroxyacylsphingosine 1−beta−galactosyltransferase activity
0
2
4
6
-log10(p-value of GO enrichment)

B

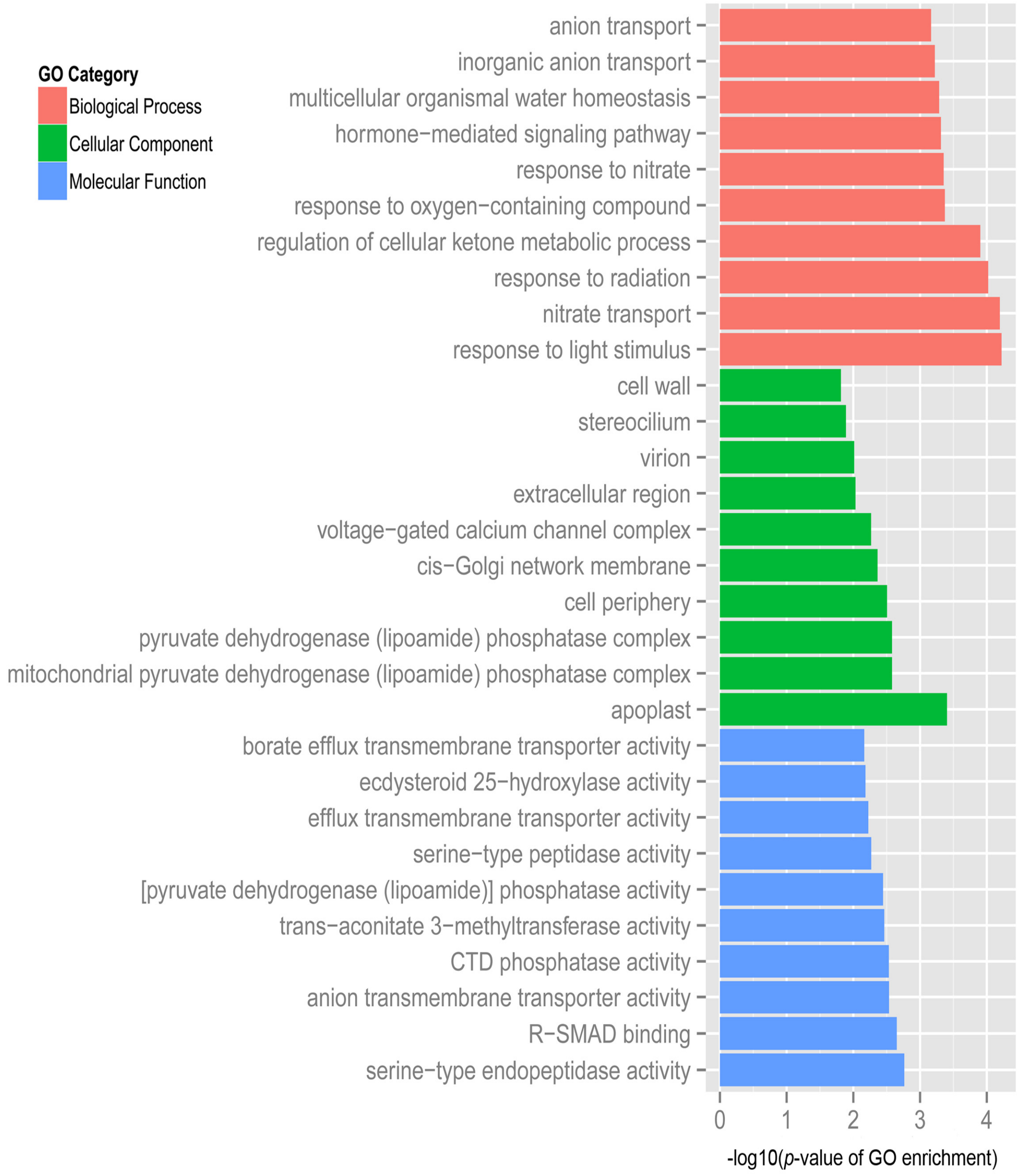

Supplementary Figure 7. Significantly enriched Gene Ontology (GO) terms (*P* < 0.05) in the resistance-specific expressed genes (RSGs) (A) and constitutively expressed genes (CEGs) (B) from Venn analysis (Table S6). GO terms in the biological process, cellular component, and molecular function domains are shown in red, green, and blue, respectively. GO terms were sorted based on *P*-values.

References

Devoto, A., Piffanelli, P., Nilsson, I., Wallin, E., Panstruga, R., von Heijne, G., et al. (1999). Topology, subcellular localization, and sequence diversity of the *Mlo* family in plants. *J. Biol. Chem.* 274, 34993-35004. doi: 10.1074/jbc.274.49.34993

Devoto, A., Hartmann, H. A., Piffanelli, P., Elliott, C., Simmons, C., Taramino, G., et al. (2003). Molecular phylogeny and evolution of the plant-specific seven-transmembrane MLO family. *J. Mol. Evol.* 56, 77-88. doi: 10.1007/s00239-002-2382-5

Kim, M. C., Lee, S. H., Kim, J. K., Chun, H. J., Choi, M. S., Chung, W. S., et al. (2002a). Mlo, a modulator of plant defense and cell death, is a novel calmodulin-binding protein: isolation and characterization of a rice *Mlo* homologue. *J. Biol. Chem.* 277, 19304-19314. doi: 10.1074/jbc.M108478200

Kim, M. C., Panstruga, R., Elliott, C., Müller, J., Devoto, A., Yoon, H. W., et al. (2002b). Calmodulin interacts with MLO protein to regulate defence against mildew in barley. *Nature* 416, 447-451. doi: 10.1038/416447a

Panstruga, R. (2005b). Discovery of novel conserved peptide domains by ortholog comparison within plant multi-protein families. *Plant Mol. Biol.* 59, 485-500. doi: 10.1007/s11103-005-0353-0
